# Comparative study on the diversity and abundance of bacterial composition in four non-biting synanthropic flies from The Gambia and China

**DOI:** 10.1186/s12917-026-05620-7

**Published:** 2026-06-15

**Authors:** Binta J.J Jallow, Afito Luciano, Mandie Liu, Yuting Ma, Yuxin Huo, Sha Tan, JingJing Huang, Jifeng Cai, Fanming Meng

**Affiliations:** 1 Department of Parasitology, School of Basic Medical Sciences, Central South University, Changsha, 410013 Hunan China; 2 Department of Forensic Medicine, School of Basic Medical Sciences, Central South University, Changsha, 410013 Hunan China; 3 Department of Forensic Medicine, Key Laboratory of Forensic Medicine, School of Basic Medical Sciences, Xinjiang Medical University, Urumqi, 830017 Xinjiang China; 4 Xinjiang Key Laboratory of Molecular Biology for Endemic Diseases, Urumqi, 830017 Xinjiang China; 5 Department of Science, University of Education, Brikama Campus, Brikama City, The Gambia; 6 Department of Agricultural and Biological Chemistry, Graduate School of Environmental, Life, Natural Science and Technology, Okayama University, Okayama City, Japan

**Keywords:** Non-biting synanthropic flies, KMC, Changsha, Illumina NovaSeq, 16S rDNA

## Abstract

**Background:**

Non-biting synanthropic flies from the order Diptera have long been implicated as mechanical vectors to several human and animal pathogens, yet their microbiome variations across different regions remain poorly understood. Here, we compared the abundance of gut microbial composition in four species (*Chrysomya megacephala*, *Lucilia cuprina*, *Musca domestica*, and *Physiphora clausa* of non-biting synanthropic flies from two countries (The Gambia and China), providing insights into the potential pathogenic bacterial taxa they harbor. This study was conducted in Kanifing Municipal Council, The Gambia, and Changsha city, China, with sampling occurring between August and November 2023. Illumina NovaSeq6000 sequencing was used to amplify the V3-V4 region of the 16S rDNA from the midgut of these flies, which were pooled (*n* = 20 per sample) into 21 samples comprising *Chrysomya megacephala*,* Lucilia cuprina*,* Musca domestica* (from both cities), and *Physiphora clausa* (from KMC). Alpha and beta diversity indices were used to compare bacterial composition in the midguts of these flies. All bioinformatics and statistical analyses were performed using the BMKCloud online platform (http://www.biocloud.net).

**Results:**

Taxonomic classification was annotated into 2 kingdoms, 44 phyla, and 2,379 species. Proteobacteria (41.14%) and Firmicutes (39.51%) dominated across all the samples. Significant geographic differences were observed. Shannon Alpha diversity analyses differed significantly between countries (*P* = 0.023), and Bray-Curtis-based PERMANOVA confirmed distinct bacterial compositions (R²=0.201, *P* = 0.001). Gambian flies harbored more *Wohlfahrtiimonas chitiniclastica* (13.79%), while Changsha samples contained higher *Pseudomonas* (3.99%). To the best of our knowledge, this is the first description of the gut microbiome of *P. clausa*, which was dominated by *Corynebacterium* (15.82%). These geographic and species-specific patterns highlight flies as reservoirs for regionally relevant pathogens.

**Conclusion:**

This study highlights the geographic variability in gut microbiota of non-biting synanthropic flies as potential carriers of regionally relevant bacterial taxa and motivates further investigation into fly-borne pathogen transmission in both regions. This study also provides, to the best of our knowledge, the first description of the gut microbiome of *P. clausa*.

**Supplementary Information:**

The online version contains supplementary material available at 10.1186/s12917-026-05620-7.

## Background

Synanthropic flies are insects that live in close association with humans, animals, and their environments, where they play important ecological and public health roles [1, 2]. These flies may be biting or non-biting, with the latter predominantly belonging to the families Calliphoridae, Muscidae, and Sarcophagidae [3]. More than 300 species of synanthropic flies have been documented worldwide, and many of which harbor diverse microorganisms on their body surfaces and within their digestive tract, making them potential mechanical vectors to pathogens [4]. Among the most common non-biting synanthropic flies reported are the *Musca domestica*, *Chrysomya megacephala*, and *Lucilia cuprina*, which possess sponging mouthparts adapted for feeding on a wide range of substrates, including decaying organic matter and human food [5, 6].

Non-biting synanthropic flies have long been implicated as mechanical vectors of microorganisms of medical, forensic, and veterinary significance, owing to their close contact with microbe-rich substrates and their frequent movement between contaminated environments and human or animal food [7, 8]. Larval stages of these flies have been found to cause myiasis in humans [9, 10]. Their life cycles and feeding behaviors frequently bring them into contact with microbe-rich environments such as animal manure, carrion, refuse, and decomposing organic matter, facilitating the dissemination of diverse microorganisms [3, 11–13]. During feeding and movement, flies can transfer pathogens via contaminated legs, mouthparts, body hairs, regurgitated gut contents, or feces, thereby contaminating food, surfaces, and wounds [14]. Consequently, a broad range of pathogens, including bacteria [3], parasites [15], fungi [16], and viruses [17] are known to be mechanically transmitted by non-biting synanthropic flies [3, 13, 18–21].

Increasing evidence suggests that the gut, particularly the midgut, serves as a more stable and diverse reservoir of microbial communities than the external body surfaces of flies [20]. While surface-associated microbes may be transient and influenced by environmental contact, midgut-associated bacteria are shaped by host physiology, diet, and microbial interactions, allowing for potential proliferation [21, 22]. Several studies have reported the gut microbial loads of these flies, suggesting a more reliable compartment for assessing fly-borne associated pathogens for a more contaminant-free bacterial microbiome [23–25]. Importantly, differences in gut microbiota composition have been observed among fly species, reflecting variations in feeding habits, habitat preferences, and ecological niches [23–25].

Despite growing interest in fly-associated microbiomes, most studies have focused on single fly species [26] or restricted geographic locations, limiting our understanding of how microbial communities vary across species and regions. Comparative studies examining multiple non-biting synanthropic fly species across distinct geographic and environmental settings remain scarce, particularly across countries with contrasting environmental and sanitation conditions. This study aimed to compare the midgut bacterial composition of four non-biting synanthropic fly species collected from KMC and Changsha, two cities that differ in sanitation infrastructure and waste management practices, providing insights into species- and geography-associated variation in microbial composition.

## Results

### Taxonomic Annotation

The bacterial 16S rDNA gene was analyzed from samples of *Chrysomya megacephala*, *Musca domestica*, *Lucilia cuprina*, and *P. clausa*. As shown in supplementary file_1_Table 1, *C. megacephala*, *M. domestica*, and *L. cuprina* each constituted six samples (3 samples from each city), amounting to 18 out of the 21 samples. Each of these three fly species had three replicates, three months apart, in both cities. Each fly species had one sample each month (fly midgut). Three *P. clausa* samples, NDS1, NDS2, and NDS3 from the KMC, were also included in the study, bringing the total to 21 samples.

A total of 2,593,693 high-quality reads were obtained from the 21 samples after quality checks and were then grouped into operational taxonomic units (OTUs). The OTUs were annotated into 2 kingdoms, 44 phyla, 120 classes, 326 orders, 706 families, 1,681 genera, and 2,379 species. The species distribution histogram shows the proportional representation of the top 10 organisms from each phylum, class, genus, and species across the four species. The other extremely low (< 1%) bacterial genera or species were consolidated into a collective category labeled as “OTHERS”.

After fly species identification, three non-biting flies were selected for midgut bacterial isolation analysis due to their high abundance in both cities, while the *P. clausa* was included because it was found only in the KMC (Gambia) samples, and no previous studies have reported on the bacterial composition of this fly. In this study, four non-biting synanthropic fly species were assessed, and 12,529 OTUs were detected. The bacterial taxa from the Changsha samples from all three species (*C. megacephala*, *L. cuprina*, *M. domestica*) showed a notably higher OTU abundance compared to the KMC samples. The Changsha samples collectively represented 63.93% of the total detected OTUs, in contrast to the lower OTU representation from the KMC samples. *L. cuprina* had the highest percentage of OTUs at 46.88%, followed by *M. domestica* at 43.5%, and *C. megacephala* at 23.4%. Bacterial taxa from the *P. clausa* accounted for 12.27% of the detected OTUs.

**Fig. 1:**
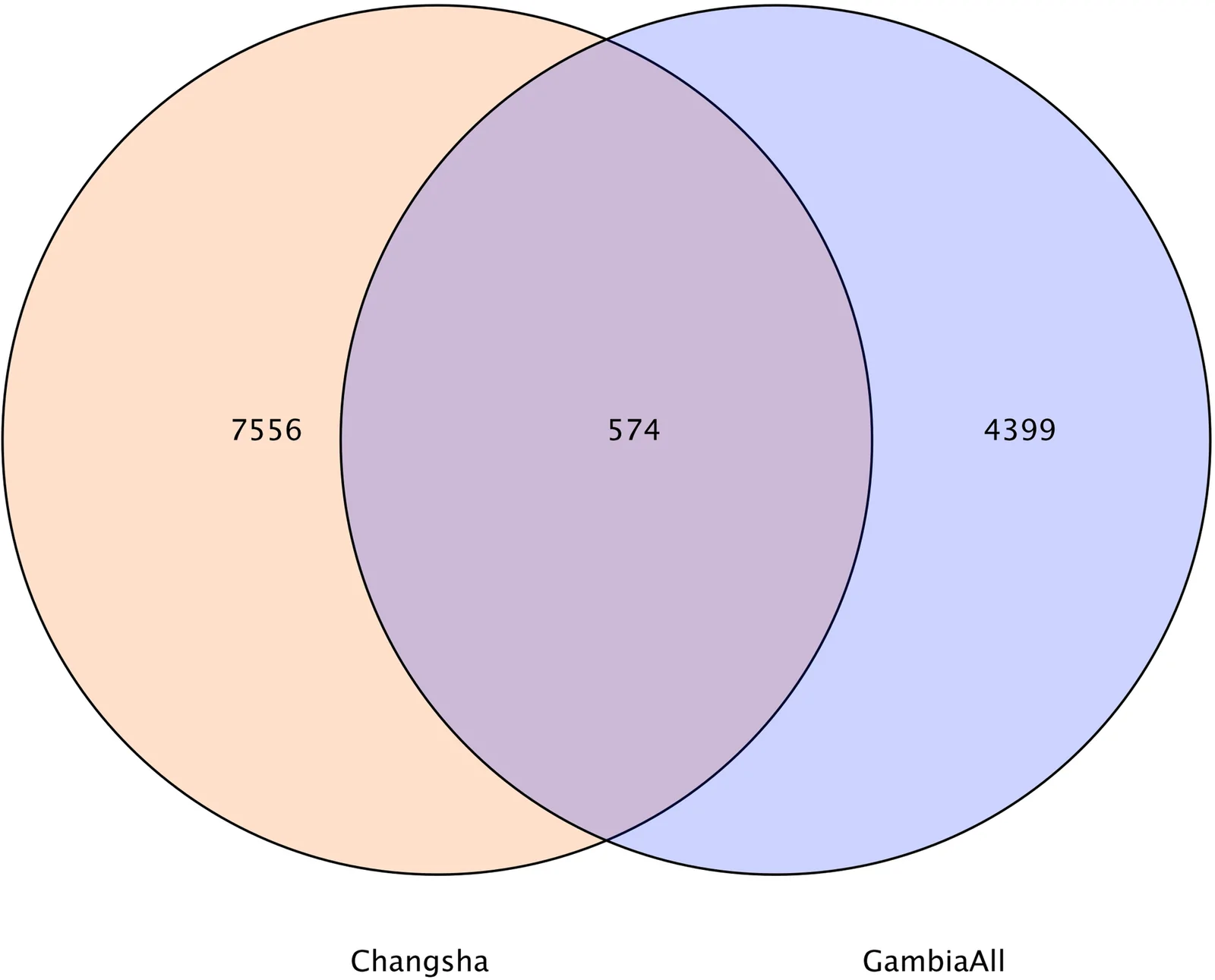
Venn diagram of OTU-level classification between all Changsha and KMC samples

**Fig. 2:**
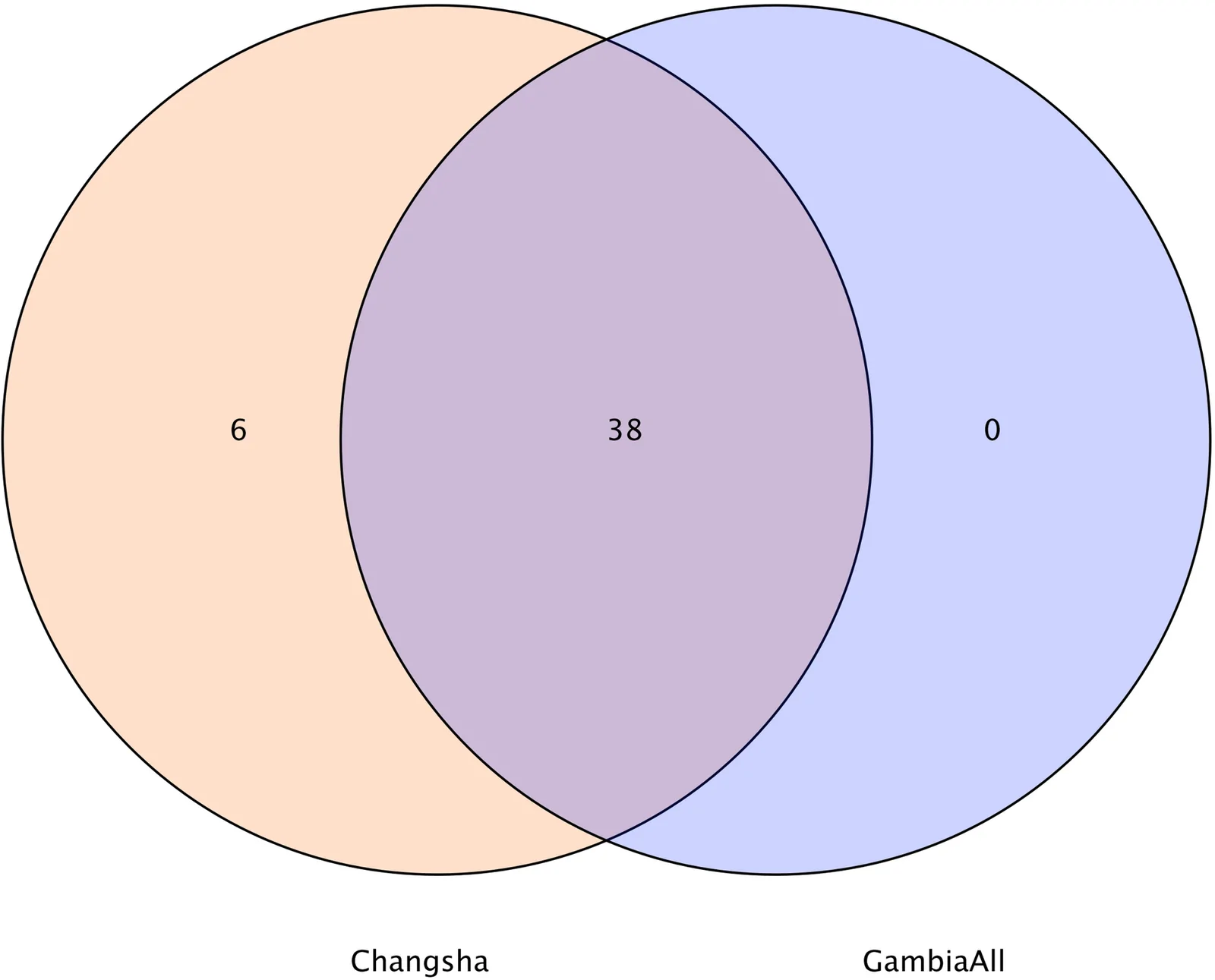
Venn diagram of phylum-level classification between all Changsha and KMC samples

**Fig. 3:**
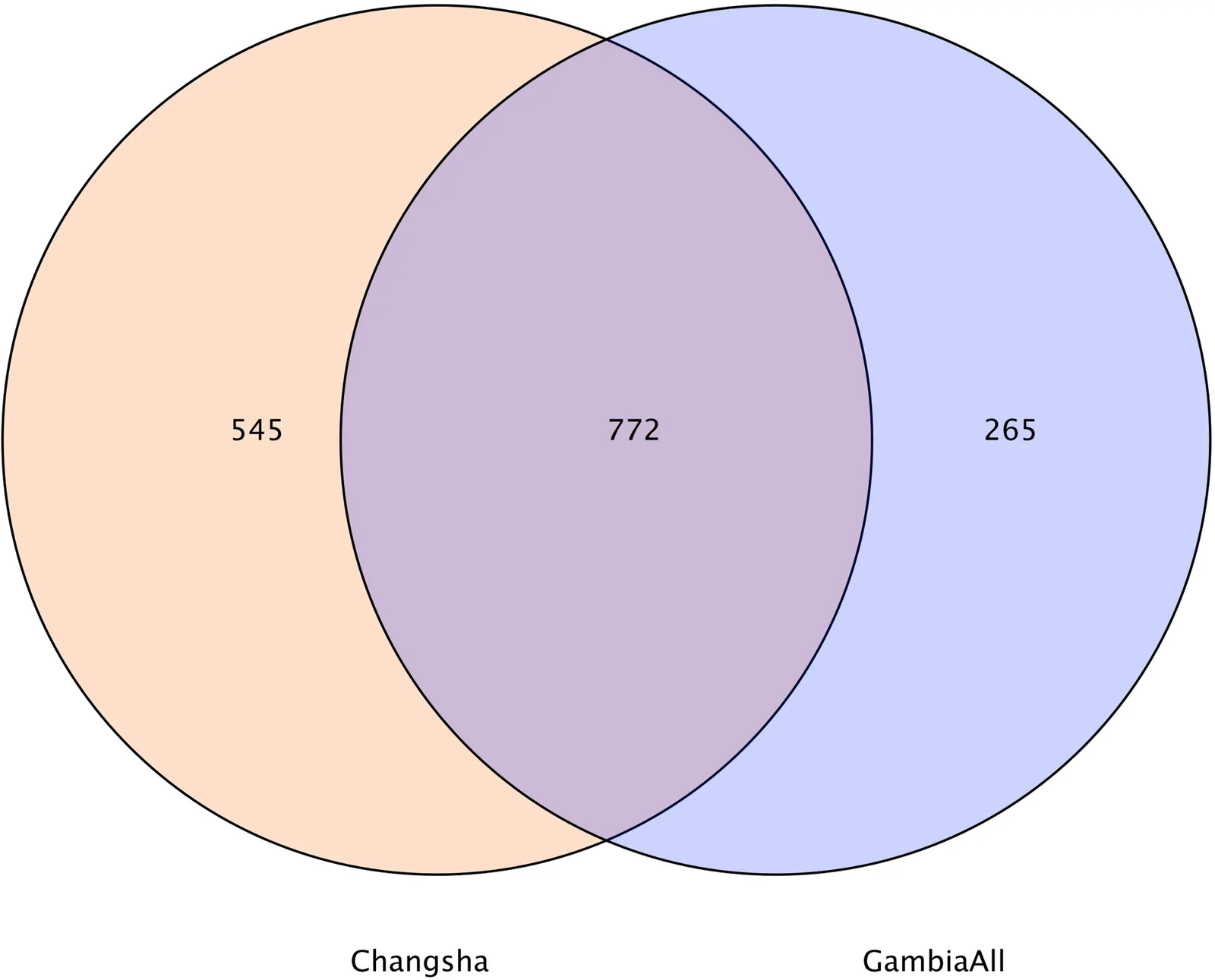
Venn diagram of genus-level classification between all Changsha samples and KMC samples

Additionally, Changsha samples recorded 7,556 unique OTUs while KMC samples recorded 4,399 unique OTUs. Samples from both cities recorded 574 shared OTUs (Figure 1). At the phylum level, Changsha recorded 6 unique OTUs, while KMC samples had no unique OTUs, and 38 shared OTUs existed between the two cities (Fig. 2). Similarly, at the genus level, Changsha samples recorded 545 unique OTUs, while KMC samples recorded 265 unique OTUs and shared 772 OTUs (Fig. 3).

**Fig. 4:**
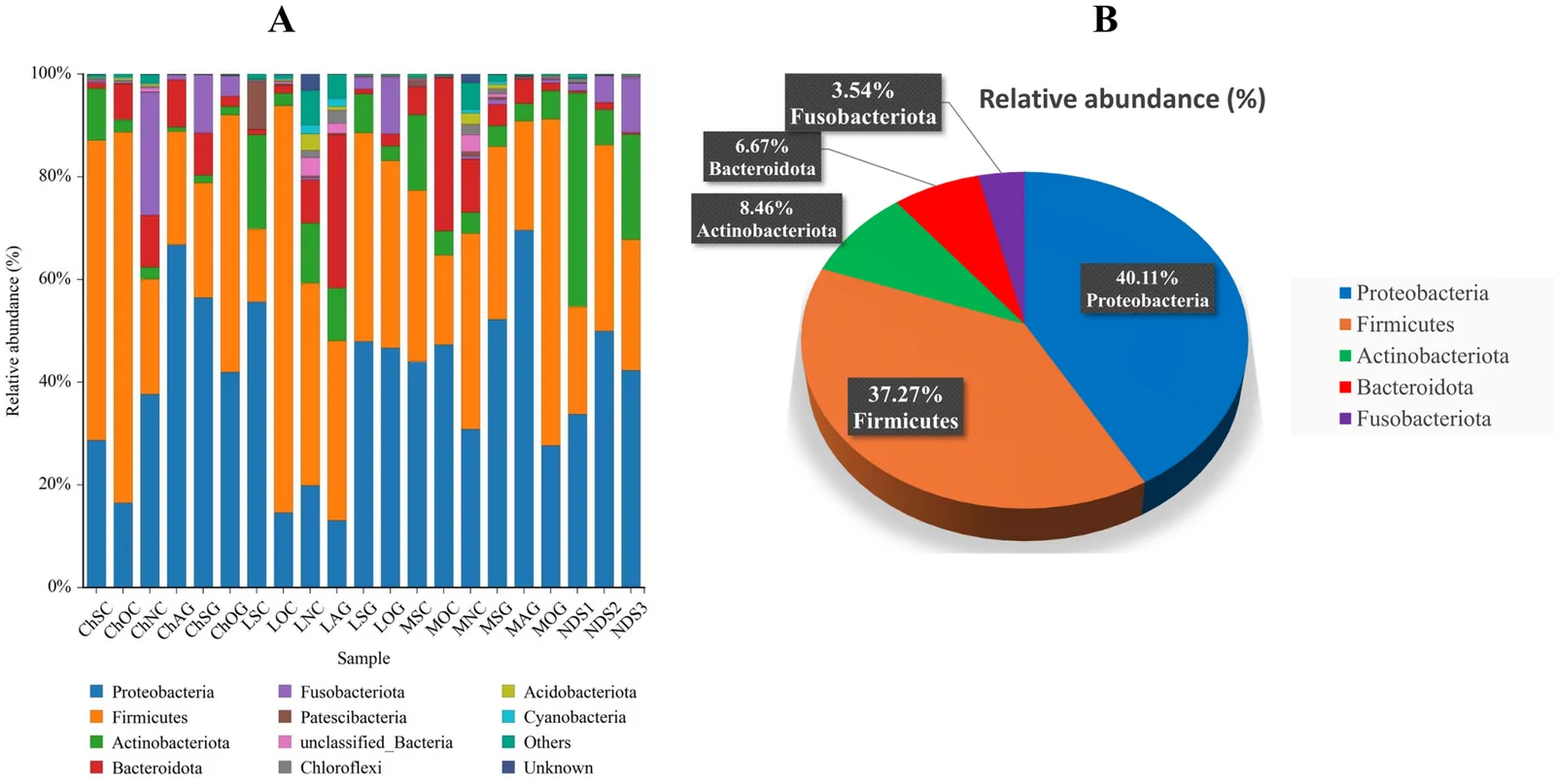
**AB**: Relative abundance (%) of bacterial taxa at phylum levels across all 21 samples from KMC and Changsha. **A** Phylum-level relative abundance (%) of the top ten bacterial taxa across all samples. **B** Relative abundances (%) of the top five phylum-level level bacterial taxa across all samples

**Fig. 5:**
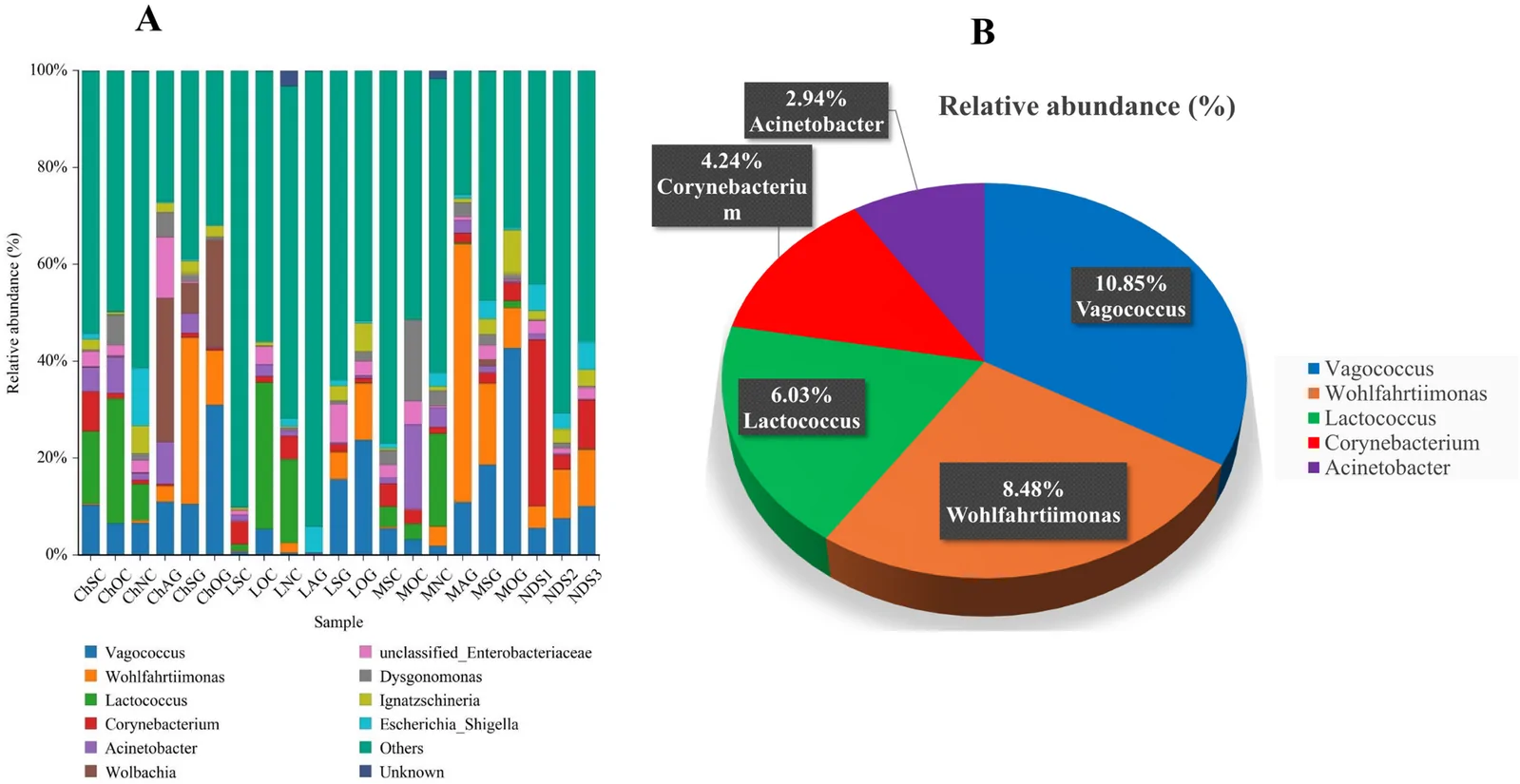
**AB**: Relative abundance (%) of bacterial taxa at genus-levels across all 21 fly midgut samples from KMC and Changsha. **A** Genus-level relative abundance (%) of the top ten bacterial taxa across individual samples. **B** Relative abundances (%) of the top five genus-level bacterial taxa across all samples

The overall most abundant bacterial taxa at the phylum level across all samples (both cities) were Proteobacteria (40.11%) and Firmicutes (37.27%), followed by Actinobacteroidota (8.46%) and Bacteroidota (6.67%) (Fig. 4A, B). Extremely low percentage bacterial phyla were also detected. At the family level, Wohlfahrtiimonadaceae (12.15%) was the most represented across all samples, followed by Vagococcaceae (10.85%) and Streptococcaceae (6.82%). Of the identified genera, *Vagococcus* (10.85%) and *Wohlfahrtiimonas* (8.48%) were the most abundant bacterial taxa. This was followed by *Lactococcus* (6.03%) and *Corynebacterium* (4.24%). Low-abundant genera included *Acinetobacter* (2.94%), *Wolbachia* (2.85%), unclassified *Enterobacteriaceae* (2.60%), *Dysgonomonas* (2.34%), *Ignatzschineria* (2.30%), and *Escherichia-Shigella* (2.24%). Most of these bacterial genera are known to harbor potential bacterial pathogens of humans and animals. Extremely low-abundant taxa “Others” accounted for 54.83% and each reported < 1% (Fig. 5A, B) (Supplementary file 2).

### Species-specific bacterial composition and abundance

#### C. megacephala samples

**Fig. 6:**
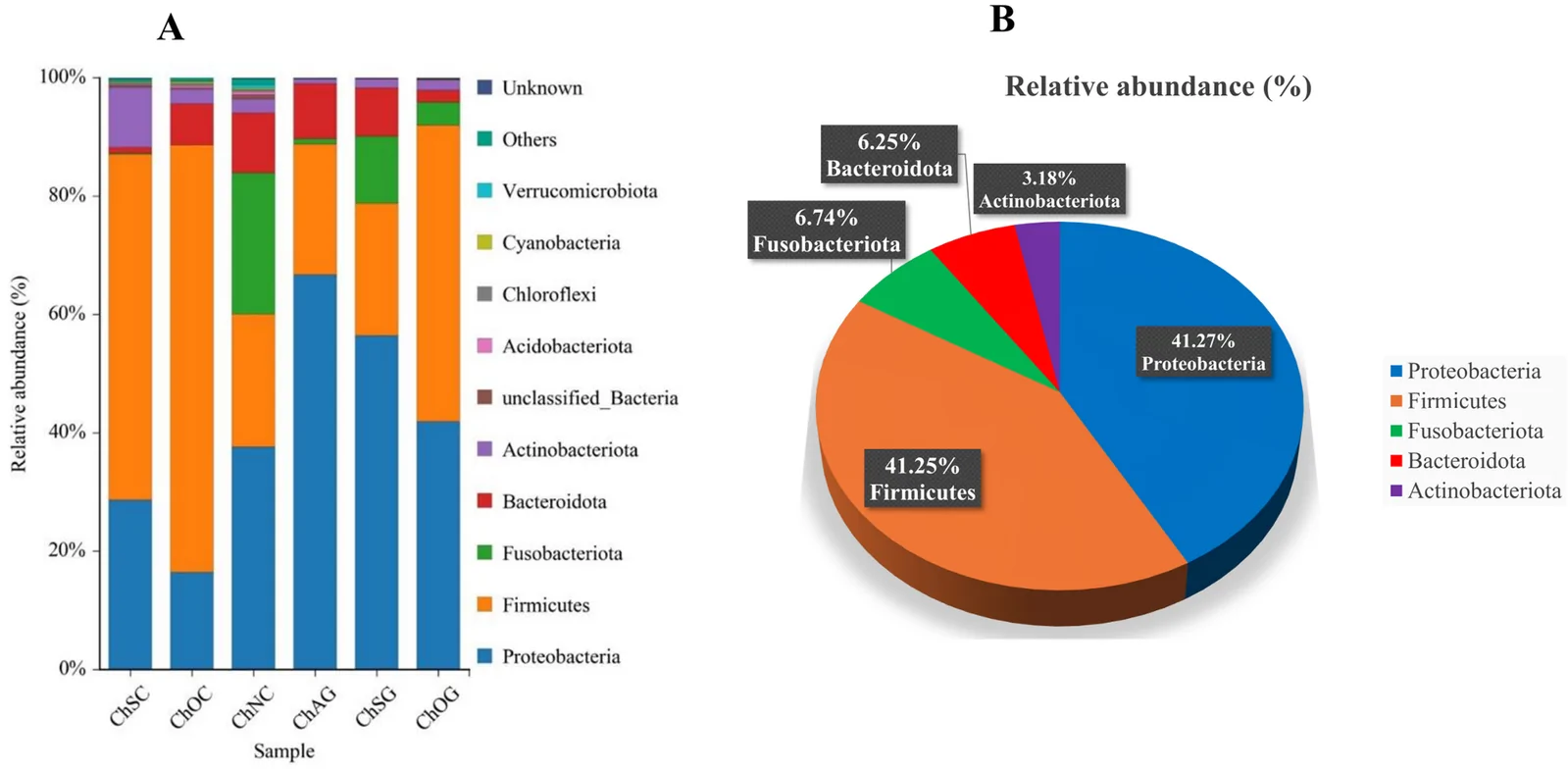
**A**-**B**: Relative abundance (%) of bacterial taxa at the phylum levels in *Chrysomya megacephala* midgut samples from KMC and Changsha. **A** Phylum-level relative abundance (%) of the top ten bacterial taxa across individual of *C. Megacephala* samples. **B** phylum-level relative abundances (%) of the top five bacterial taxa across individual *C. megacephala* samples

Significant differences in bacterial composition at the phylum and genus levels were observed within *C. megacephala* samples from Changsha and KMC. The most abundant bacterial phyla were Proteobacteria (41.26%) which were higher in KMC samples, and Firmicutes (41.25%) which were higher in Changsha samples. Other low-abundant bacterial taxa encountered were Fusobacteria (6.73%), Bacteroidota (6.25%), and Actinobacteriota (3.18%) (Fig. 6A, B) which were reported in samples from both cities.

At the family level, Wohlfahrtiimonadaceae (13.27%) and Vagococcaceae (12.66%) were the most abundant families, followed by Anaplasmataceae (9.76%), Streptococcaceae (8.78%), Fusobacteriaceae (6.70%), and Lactobacillaceae (6.38%). Family Wohlfahrtiimonadaceae, Vagococcaceae, and Anaplasmataceae were relatively predominant in KMC samples compared with Changsha replicates. Conversely, Streptococcaceae was prominently higher in the latter. Fusobacteriaceae, Lactobacillaceae, and Enterobacteriaceae were detected in all replicates in varying low abundances.

**Fig. 7:**
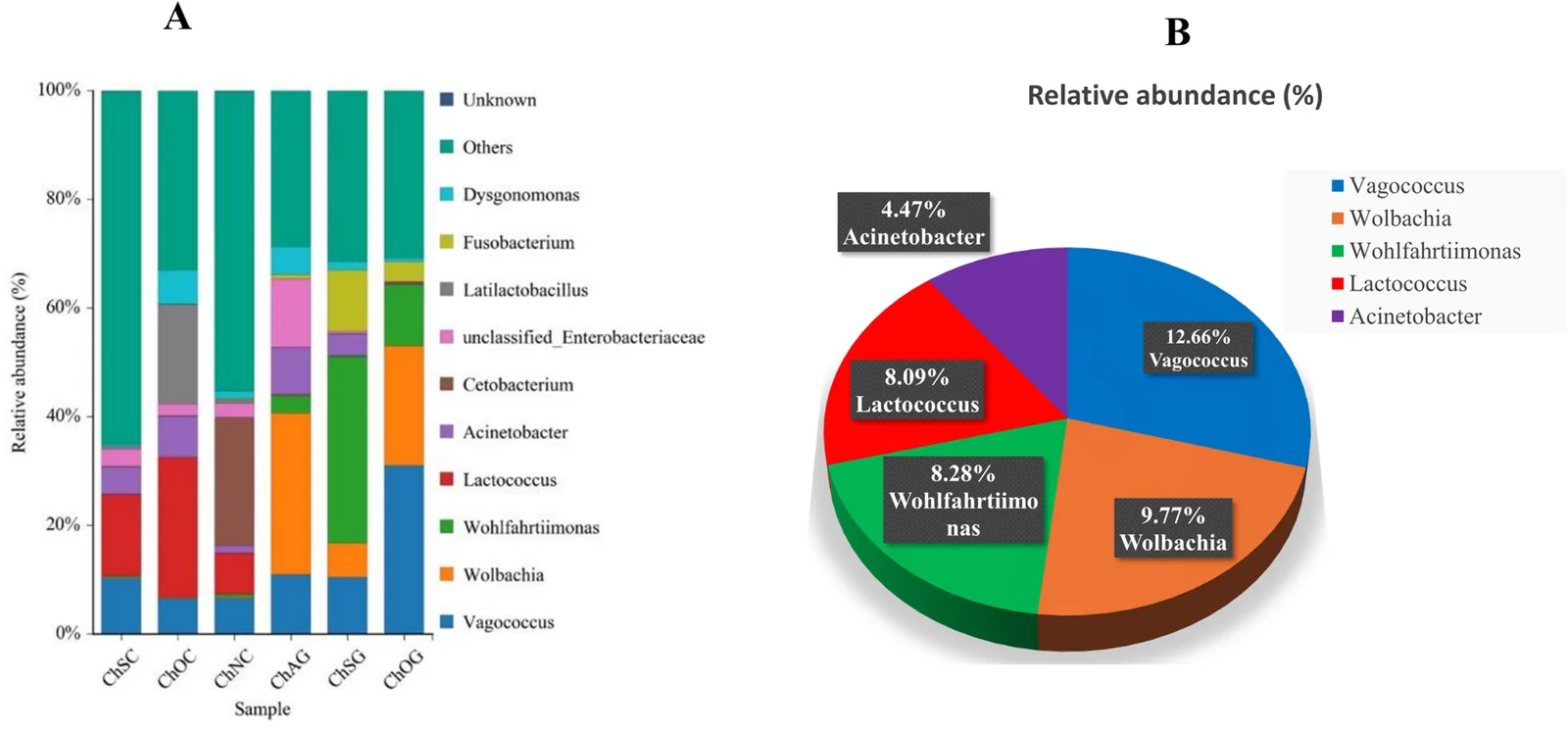
**A**-**B**: Relative abundance (%) of bacterial taxa at the phylum and genus levels in *Chrysomya megacephala* midgut samples from KMC and Changsha. **A** Relative abundances (%) of the top ten phylum-level bacterial taxa in *C. megacephala* samples from both cities. **B** Relative abundance (%) of the top five genus-level bacterial taxa in *C. megacephala* samples from both cities

Most bacterial taxa were classified up to the genus level across the *C. megacephala* samples. *Vagococcus* (12.66%) was found in all samples; *Wolbachia* (9.77%) was found only in KMC samples; *Wohlfahrtiimonas* (8.28%) was found only in KMC samples; and *Lactococcus* (8.09%) was found only in Changsha samples. These bacterial taxa recorded the highest abundances in *C. megacephala*. *Lactilactobacillus* bacteria were present in all Changsha replicates but absent from all three KMC samples. *Fusobacterium* was detected in all samples except the ChOC replicate, suggesting that the month of collection influenced the bacterial composition of the samples. *Dysgonomonas* and other bacterial genera were present in all six samples in varying abundances (Fig. 7A, B) (Supplementary file 3).

#### L. cuprina samples

**Fig. 8:**
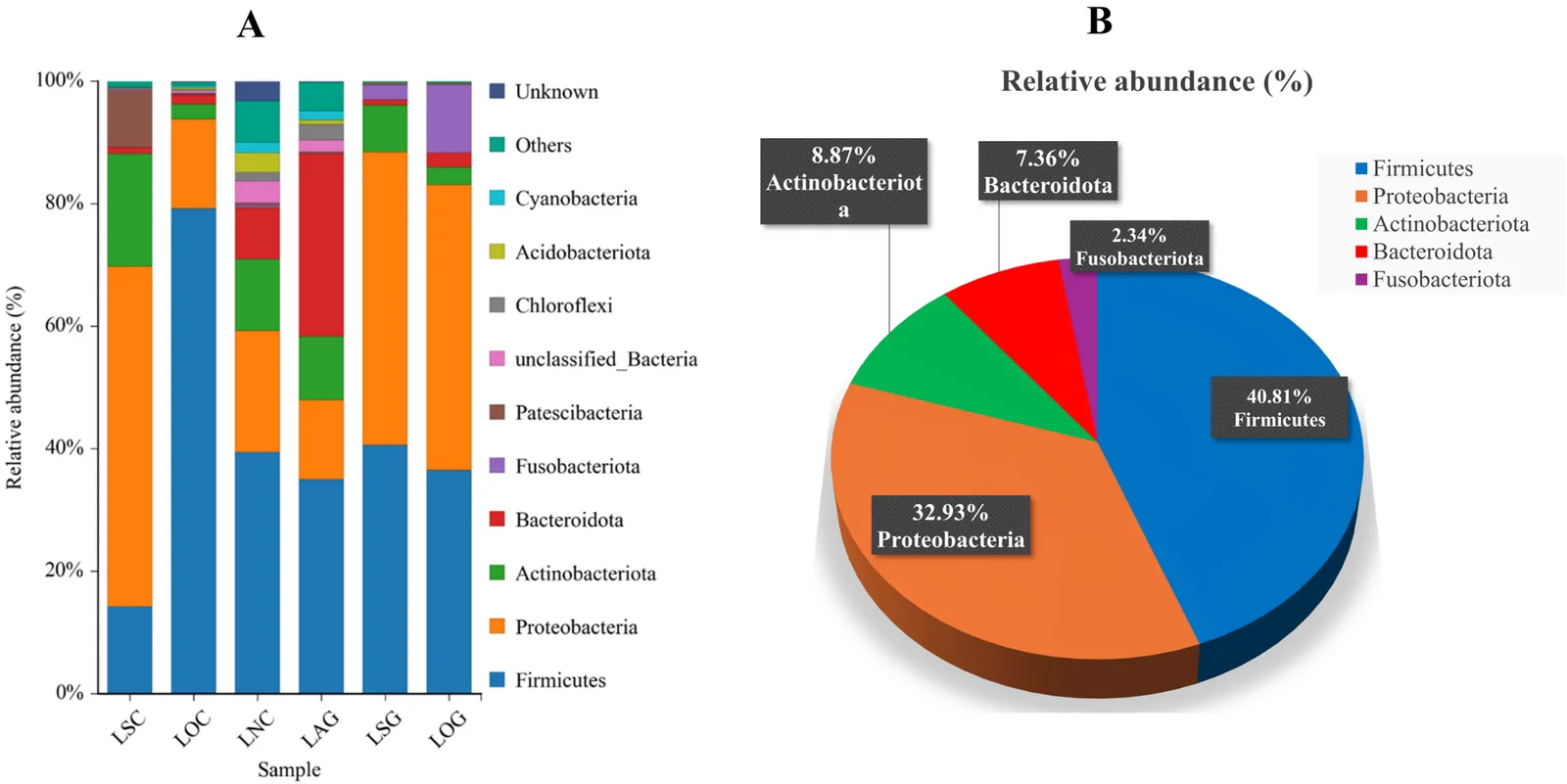
**A**-**B**: Relative abundance (%) of bacterial taxa at the phylum levels in *Lucilia cuprina* midgut samples from KMC and Changsha. **A** Phylum-level relative abundance (%) of the top ten bacterial taxa across individual *L. cuprina* samples. **B** Phylum-level relative abundance (%) of the top five bacterial taxa across individual *L. cuprina* samples

Phylum Firmicutes and Proteobacteria accounted for approximately 74% of the total bacterial OTU of the *L. cuprina* samples from both cities. However, we observed significant variations among the six samples across the phyla. For example, LOC, LSG, and LNC recorded the highest abundances in the phylum Firmicutes compared to the other three samples. Unlike the *C. megacephala* samples, the *L. cuprina* samples show no significant variation between the two cities. Fusobacteriota was < 1% in all three Changsha samples, absent in the LAG sample, and relatively higher in LOG and LSG. Probacterium phyla were higher in the LSC sample (9.23%) and were < 1% in all the other samples (Fig. 8A, B).

At the family level, Streptococcaceae, Lactobacillaceae, Vagococcaceae, and Wohlfahrtiimonadaceae (respectively) recorded the highest abundances across the *L. cuprina* samples. The Vagococcaceae and Wohlfahrtiimonadaceae families were slightly more abundant in the KMC samples.

**Fig. 9:**
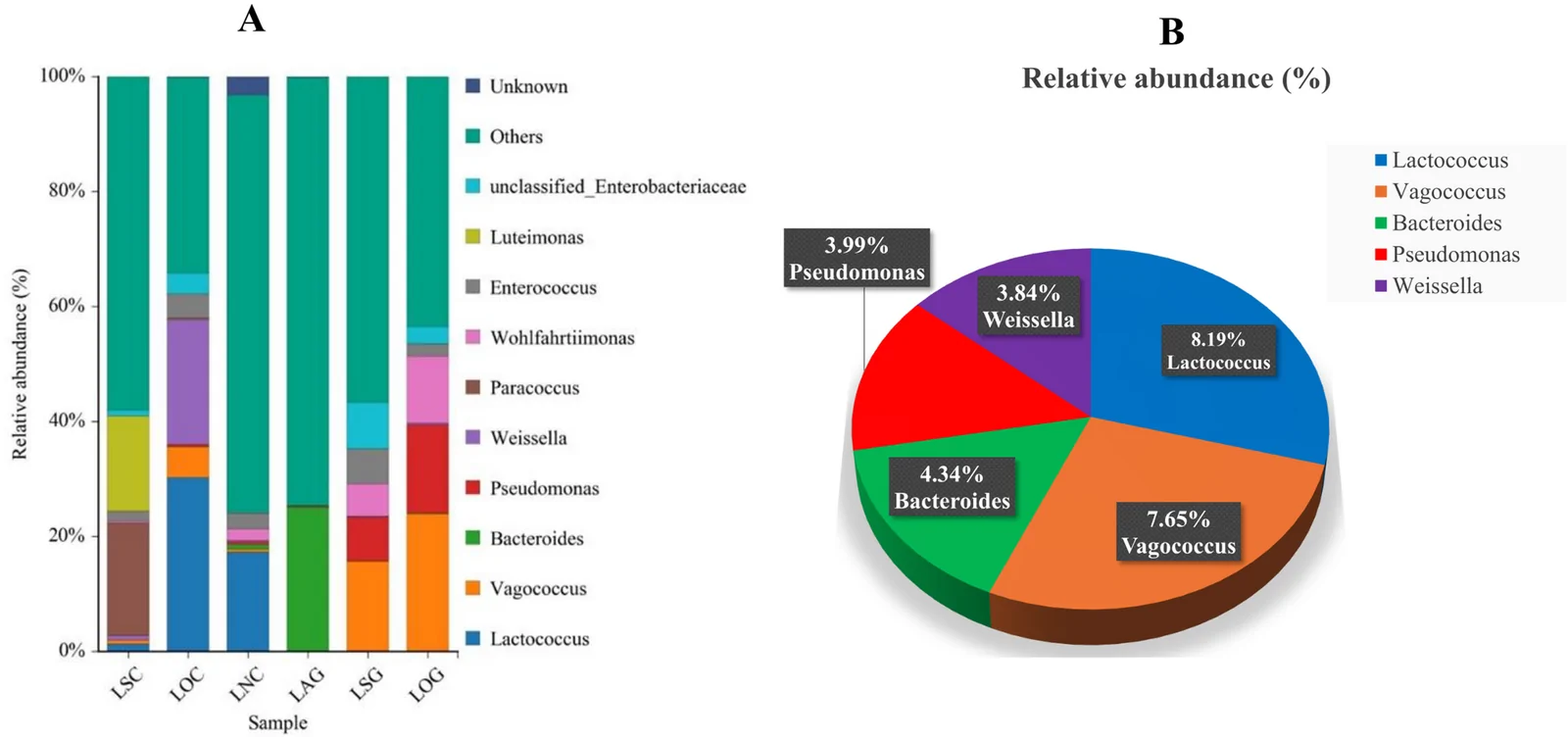
**A**-**B**: Relative abundance (%) of bacterial taxa at the phylum levels in *Lucilia cuprina* midgut samples from KMC and Changsha. **A** Relative abundances (%) of the top ten phylum-level bacterial taxa in *L. cuprina* samples from both cities. **B** Relative abundances (%) of the top five genus-level bacterial taxa in *L. cuprina* samples from both cities

*Lactococcus* (8.19%) and *Vagococcus* (7.65%) were the most abundant bacterial genera. This was followed by *Bacteroides* (4.34%), *Pseudomonas* (3.99%), *Weissella* (3.84%), *Paracoccus* (3.36%), and *Wohlfahrtiimonas* (3.26%). *Lactococcus* bacterial taxa were present only in the Changsha samples. *Vagococcus* and *Bacteroides* genera recorded higher abundances in the KMC samples. *Pseudomonas* bacterial taxa were present in all samples, but showed higher abundances in the Changsha samples. *Weissella* and *Paracoccus* were present only in the Changsha samples. On the contrary, *Wohlfahrtiimonas* taxa were highly prevalent in the KMC samples (high abundances). *Enterococcus* was detected in all samples except the LAG replicate. *Luteimonas* bacterial taxa were present in only the Changsha samples (Fig. 9A, B) (Supplementary file 4).

#### M. domestica

**Fig. 10:**
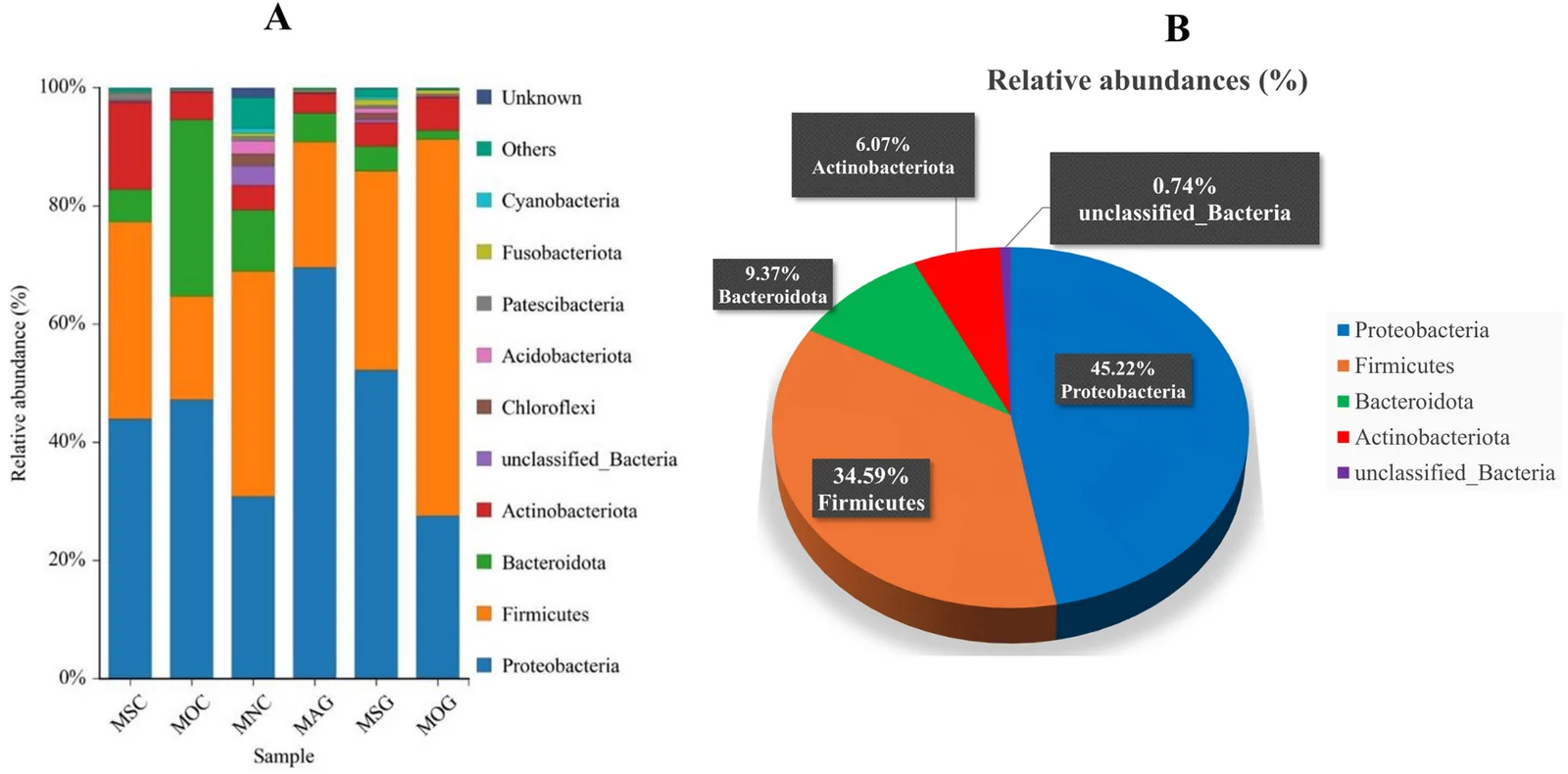
**A**-**B**: Relative abundance (%) of bacterial taxa at the phylum levels in *Musca domestica* midgut samples from KMC and Changsha. **A** Phylum-level relative abundance (%) of the top ten bacterial taxa across individual *M. domestica* samples. **B** Phylum-level relative abundance (%) of the top five bacterial taxa across individual *M. domestica* samples

Phylum Proteobacteria (45.22%) and Firmicutes (34.54%) recorded the highest relative abundances from the *M. domestica samples* from both cities. This was followed by low proportions of bacterial phyla, Bacteroidota (9.37%) and Actinobacteriota (6.07%). All the remaining phyla, Chloroflexi, Patescibacteria, Fusobacteriota, and Cyanobacteria, recorded extremely low abundances (< 2% each) (Fig. 10A, B) from samples in both cities.

Wohlfahrtiimonadaceae, Vagococcaceae, Streptococcaceae, Lactobacillaceae, Enterobacteriaceae, Moraxellaceae, and Dysgonomonadaceae were the seven most abundant bacterial families recorded from *M. domestica* across the six samples. Wohlfahrtiimonadaceae and Vagococcaceae were more abundant in the KMC samples. On the other hand, Streptococcaceae and Lactobacillaceae families were most predominant in the Changsha samples.

**Fig. 11:**
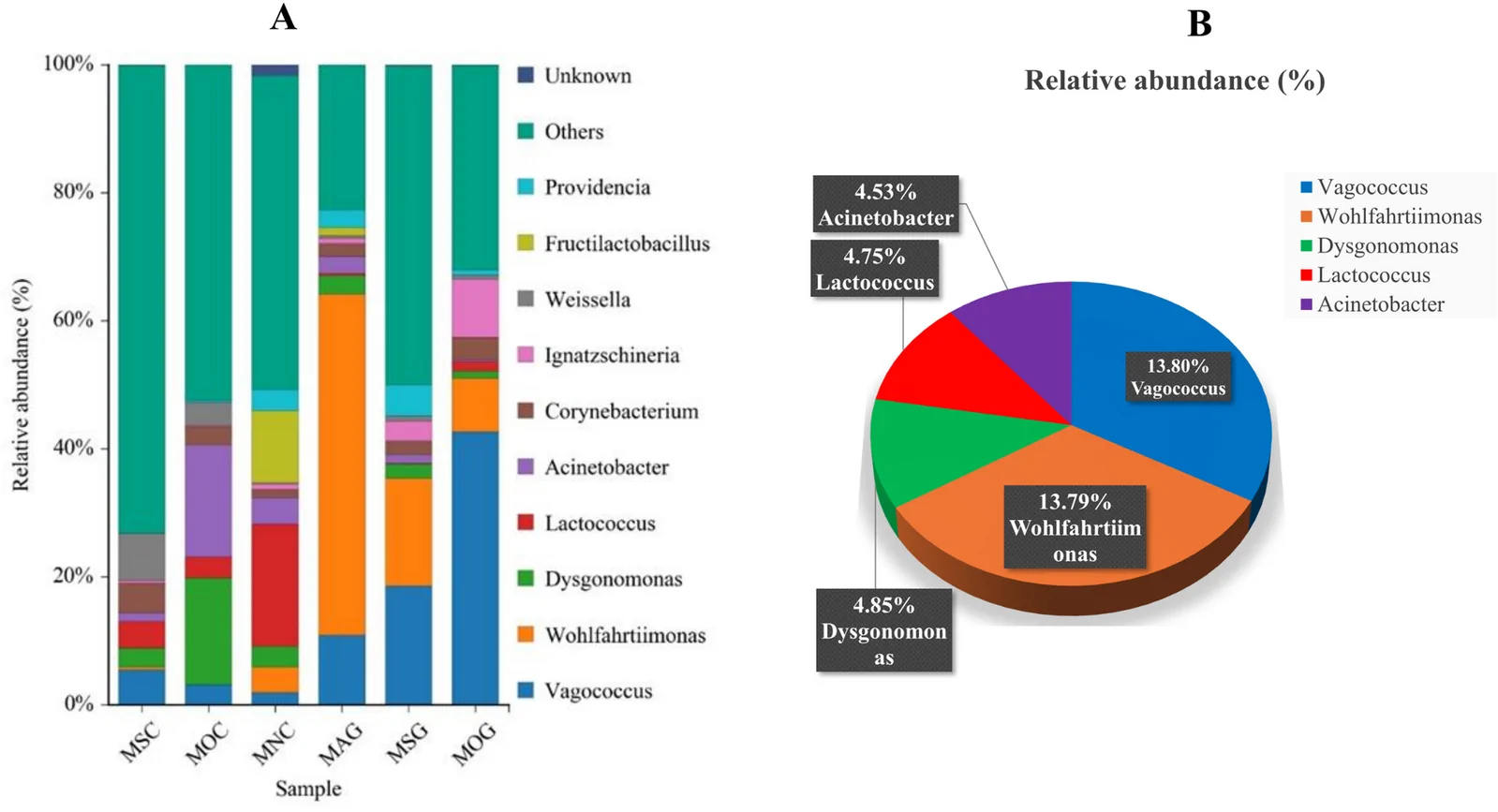
**A**-**B**: Relative abundance (%) of bacterial taxa at the genus levels in *Musca domestica* midgut samples from KMC and Changsha. **A** Relative abundances (%) of the top ten genus-level bacterial taxa in *M. domestica* samples from both cities. **B** Relative abundances (%) of the top five genus-level bacterial taxa in *M. domestica* samples from both cities

*Vagococcus* (13.80%) and *Wohlfahrtiimonas* (13.79%) were the most predominant bacterial genera and were higher in KMC samples than in Changsha samples. Conversely, the Changsha samples showed the highest bacterial dominance in the *Dysgonomonas*,* Lactococcus*, and *Acinetobacter* bacterial genera. *Weissella* and *Fructilactobacillus* bacterial taxa were higher in the Changsha samples. *Corynebacterium* and *Providencia* bacterial genera were present in all samples in similar proportions. *Ignatzschineria* bacterial taxa were present in only the KMC samples (Fig. 11A, B) (Supplementary file 5).

#### P. clausa

**Fig. 12:**
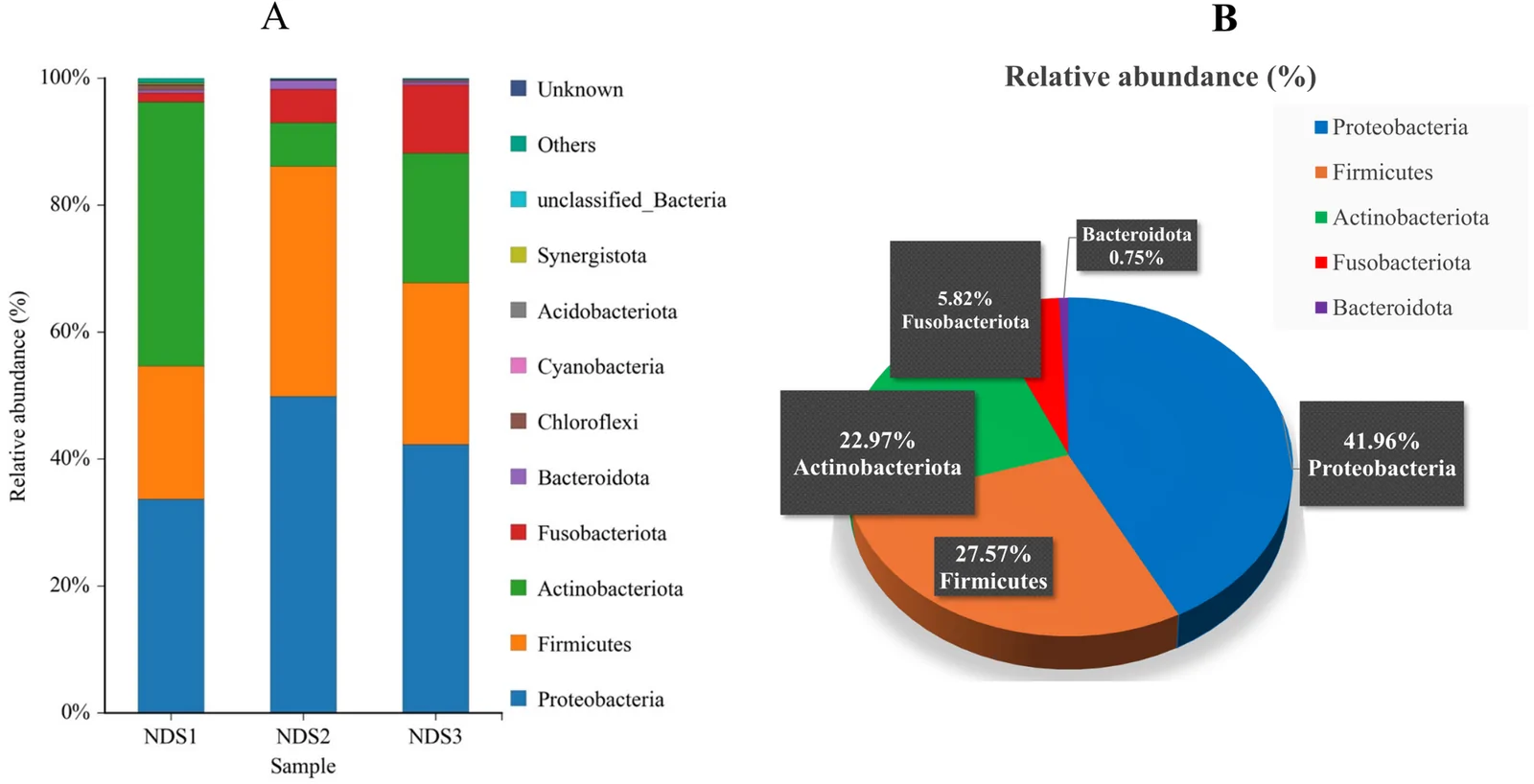
**A**-**B**: Relative abundance (%) of bacterial taxa at the phylum levels in *Physiphora clausa* from KMC. **A** Phylum-level relative abundance (%) of the top ten bacterial taxa across individual *P. clausa* samples. **B** Phylum-level relative abundance (%) of the top five bacterial taxa across individual *P. clausa* samples

Proteobacteria (41.96%), Firmicutes (27.57%), and Actinobacteria (22.97%) bacterial phyla recorded the highest relative abundances from *P. clausa* samples. This was followed by an abundant bacterial phylum, Fusobacteriota (5.82%) (Fig. 12A, B). Unlike the other three fly species, all these families—Morganellaceae, Wohlfahrtiimonadaceae, Enterobacteriaceae, Vagococcaceae, Fusobacteriaceae, Lactobacillaceae, Coriobacteriaceae, Bacillaceae, Peptostreptococcaceae from *P. clausa* were detected in very high abundance across the sample.

**Fig. 13:**
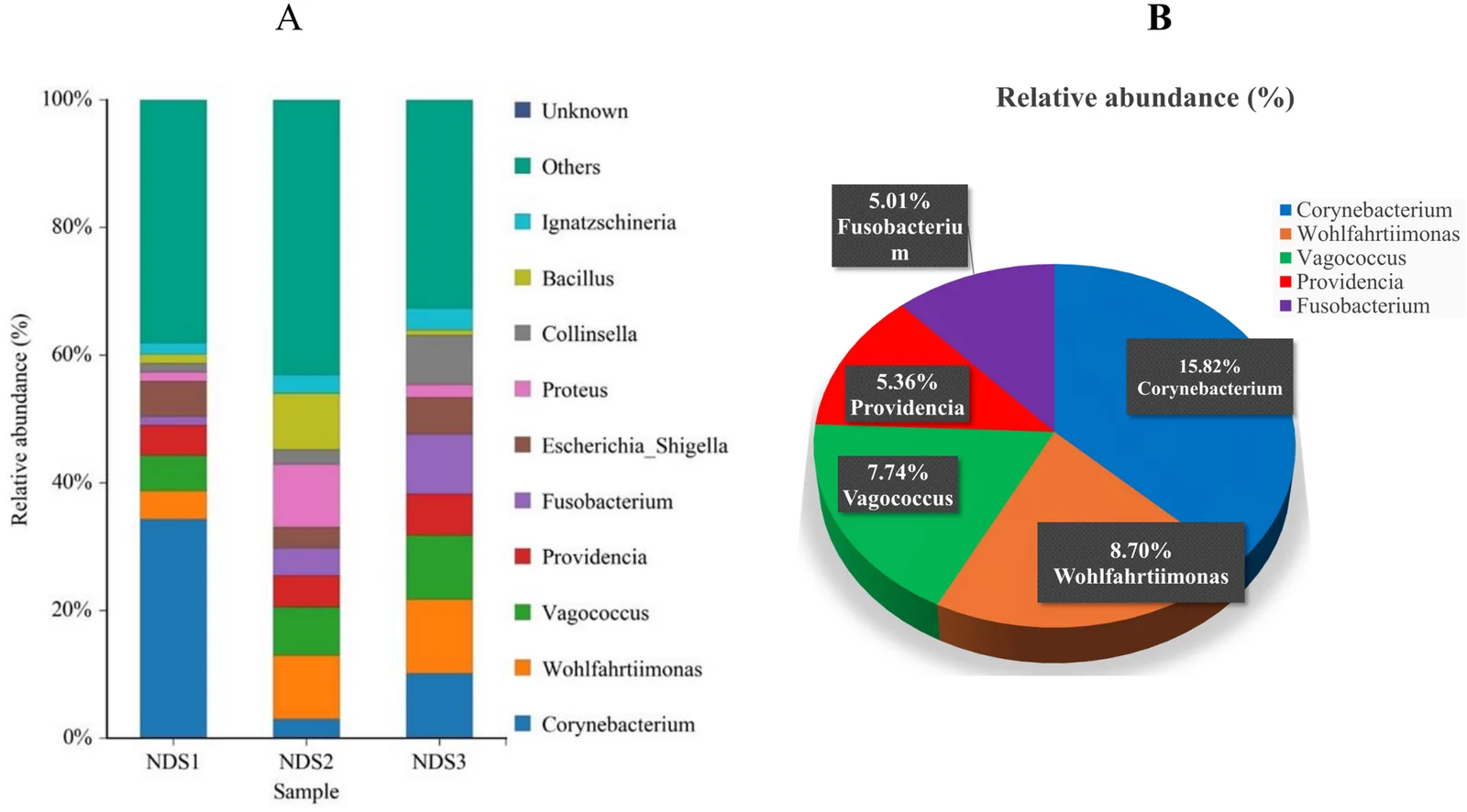
**A**-**B**: Relative abundance (%) of bacterial taxa at the phylum and genus levels in *Physiphora clausa* from KMC. **A** Relative abundances (%) of the top ten phylum-level bacterial taxa in *P. clausa* samples. **B** Relative abundances (%) of the top five genus-level bacterial taxa in P. clausa samples

*Corynebacterium* (15.82%) recorded the highest bacterial genus from the *P. clausa* samples. This was followed by low-abundant bacterial genera such as *Wohlfahrtiimonas* (8.70%), *Vagococcus* (7.74%), *Providencia* (5.36%), *Fusobacterium* (5.01%), *Escherichia-Shigella* (4.84%), *Proteus* (4.45%), *Collinsella* (3.79%), *Bacillus* (3.67%), and *Ignatzschineria* (2.71%) (Fig. 13A, B) (Supplementary file 6).

### Alpha and Beta diversity metrics

**Fig. 14:**
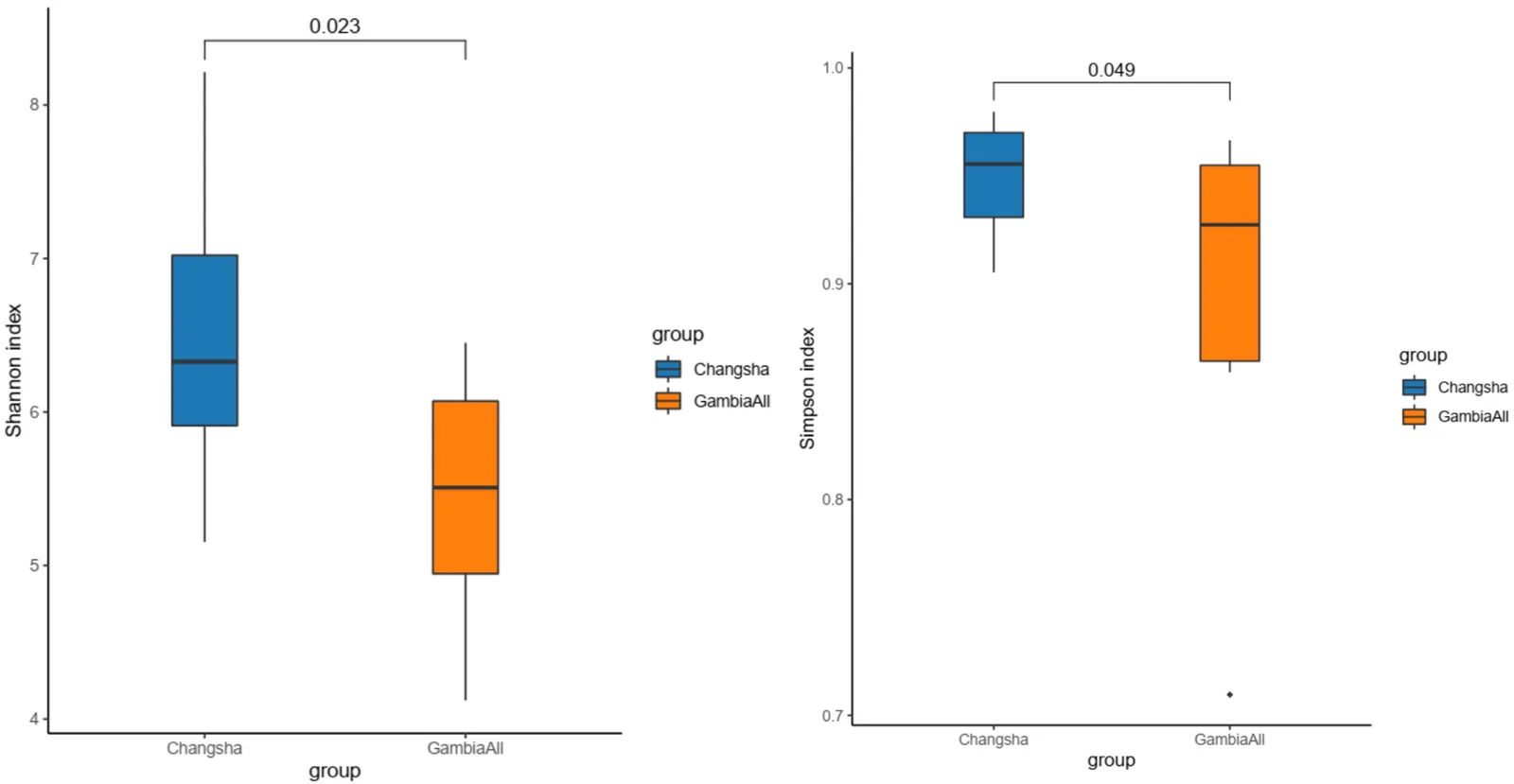
Alpha diversity indices of the midgut of synanthropic flies from both cities (*p* < 0.05). Shannon index; Wilcoxon rank-sum test (*p* < 0.023) from KMC and Changsha. Simpson index (Wilcoxon rank-sum test, *p* < 0.049) from KMC and Changsha. Beta Diversity of the midgut of non-biting synanthropic flies; Principal Component Analysis (PCoA) and PERMANOVA analysis of the midgut of four synanthropic flies from both cities, Wilcoxon rank-sum test *p* < 0.05

The richness and bacterial diversity among the midgut of four non-biting synanthropic fly samples were estimated using the Shannon and Simpson alpha diversity indices. Wilcoxon rank-sum was used to analyze the statistical significance of the data, with a significance level set at *P* < 0.05. Shannon indices of the bacterial composition of samples from Changsha were significantly different (*P* = 0.023), and the Simpson index showed the same pattern with a significant difference (*P* = 0.049) (Fig. 14). However, samples from both cities recorded a high Simpson index exceeding 0.9, indicating generally diverse and evenly distributed bacterial communities.

**Fig. 15:**
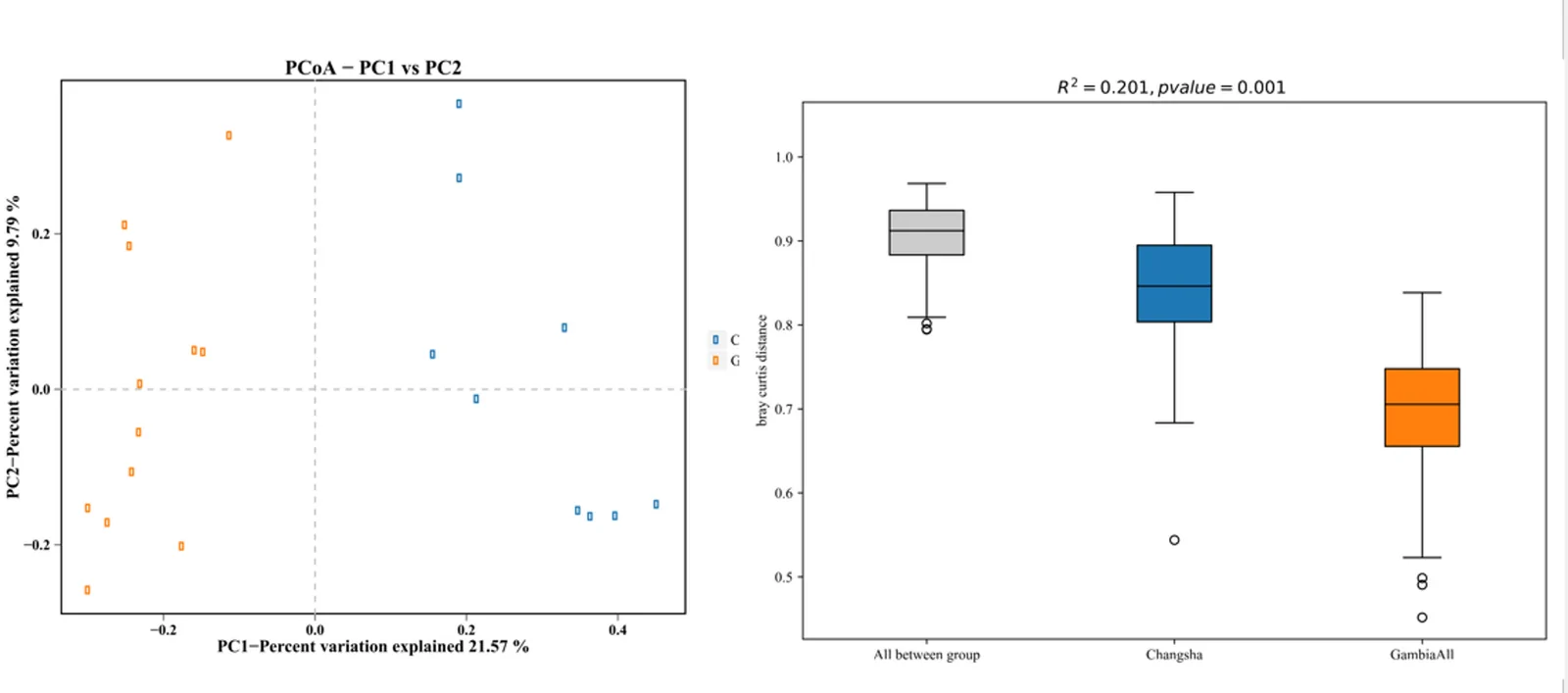
Alpha diversity indices of the midgut of synanthropic flies from both cities (*p* < 0.05). PCoA1-2 samples from the KMC and Changsha. PERMANOVA analysis of samples from KMC and Changsha (R2 = 0.201, *p* = 0.001*)

The limited variance reported by PCoA (21.57%) explained, and the dispersed clustering patterns suggest that additional environmental and ecological variables, including sanitation conditions, seasonal effects, and feeding substrates, may likely contribute to microbiome variation. Samples from both countries were dispersedly clustered. However, samples from Changsha were less clustered, indicating higher bacterial diversity, especially in the Changsha samples. The PCoA analysis using the Bray-Curtis dissimilarity metric, followed by PERMANOVA, reveals a statistically significant difference in bacterial composition between samples from the two countries, supporting the influence of geographic factors on microbial structuring. The findings of this study demonstrate that the microbial composition of Changsha city and KMC samples differed considerably across all groups (R2 = 0.201, *P* = 0.001*) (Fig. 15).

### Abundance and prevalence of potential pathogens

Potential pathogenic bacterial taxa of human and animal relevance were detected in all four fly species from both cities. Variations in bacterial abundance were largely species-dependent; however, geographic location and local environmental factors also influenced bacterial diversity and composition, although these factors were not the focus of this study.

Most detected potential pathogenic bacterial taxa were country-, month-, and species-specific. The potential bacterial pathogens recorded from KMC samples included *Wohlfahrtiimonas chitiniclastica*, *Ignatzschineria ureiclastica*, *Ignatzschineria indica*, and *Vagococcus teuberi*. The abundance of *Weissella ceti* was highly affected by the fly species (*L. cuprina)*. Erysipelothrix (*Erysipelothrix rhusiopathiae*), Flavobacteriaceae (*Myroides phaeus*), *Fusobacterium* sp. RMA1065, *Acinetobacter* sp. PP1 bacterial species were detected only in *C. megacephala* samples. Similarly, the prevalence of *Fusobacterium* sp. *Marseille P3599* was also detected in both *C. megacephala* and *L. cuprina* samples. *Corynebacterium* (*Corynebacterium ulceribovis*,* Corynebacterium humireducens*) was detected only in the *P. clausa* from the Gambia. Other important unclassified potential pathogens detected included unclassified *Enterobacter*, unclassified *Klebsiella*, and unclassified *Enterococcus* across the 21 samples, in varying proportions and at low abundances.

## Discussions

Several studies have implicated non-biting synanthropic flies as mechanical vectors to a variety of human and animal pathogens [26]. The house fly (*M. domestica*), the oriental fly (*C. megacephala*), and the Australian blowfly (*L. cuprina*) are among the most widely studied insects that live in close association with humans and have been found to carry several bacteria on their midgut [27, 28]. Their feeding and environmental settings affect the gut microbial composition. Despite these flies sharing a similar environmental niche, they have been found to differ in the bacterial composition of their gut contents [29–31]. We equally investigate the gut bacterial content from *P. clausa* (Diptera: Ulidiidae), and to the best of our knowledge, this is the first study to isolate bacteria from *P. clausa*. This is also the first bacterial isolation study to compare the bacterial composition of four different non-biting synanthropic flies from KMC and Changsha over a three-month study period, with midgut bacterial content as the main comparison factor [21]. This study was a 3-month replicate study in both countries, conducted to monitor the consistency of bacterial composition over the study duration. However, we found that bacterial composition of the same fly species shows considerable variations in the different replicate months, suggesting the month of fly species collection largely influences the bacterial composition of a fly species. While this study did not primarily focus on the seasonal variation of bacterial composition, as reported elsewhere, it was not the focus of this study [36], the pattern discussed aims to shed light on the differences in fly midgut bacterial composition associated with species and geography.

### Bacterial composition analysis

Proteobacteria and Firmicutes recorded the highest relative abundances in samples from both cities. Bacteroidota, Actinobacteriota, and Fusobacteriota phyla were present in all samples; however, they were recorded in extremely low abundances. These phyla were also dominant phyla in previous studies of the gut bacterial composition in *C. megacephala*, which reported Proteobacteria, Firmicutes, and Bacteroidetes to be the most predominant phyla, and this is similar to the results from this study [25, 32]. This could be due to their similar ecological niches. Even though the bacterial composition of *P. clausa* is unknown, results from our study revealed a bacterial composition at the phylum level similar to that reported in other synanthropic fly studies, for example, the housefly [33]. The high abundance of the phyla Proteobacteria and Firmicutes in flies could be associated with their roles in decomposing organic matter [34].

Despite all the studied samples having similar bacterial composition at the phylum taxonomic level, interesting variations were detected at other taxonomic levels, such as family, genus, and species, with varying relative abundances, which is crucial to our findings. For example, the most prevalent genus from all the samples in this study is *Corynebacterium*, and this genus was detected in very high abundances from the *P. clausa* samples from KMC, as reported in houseflies elsewhere [33]. *Vagococcus* and *Wohlfahrtiimonas* recorded the highest abundances from *C. megacephala* and *M. domestica* samples in this present study, and they were most predominantly detected from the KMC samples. These results might be associated with the widely shared microbiome that exists between *C. megacephala* and *M. domestica* due to shared feeding sources [31], and may also reflect the higher availability of decomposing organic substrates at KMC collection site where these bacterial taxa are highly enriched.

Contrary to *C. megacephala* and *M. domestica*, the *Lactococcus* genus was the most prevalent bacterial taxon among *L. cuprina* samples, in contrast to other fly samples from this study. *Ignatzschineria* genus was detected only in *M. domestica* and *P. clausa* samples and was most dominant in the KMC samples. The *Wolbachia* genus was present on *C. megacephala* samples and in very high abundance (9.77%), especially from the Changsha samples, and this bacterial taxon has been previously reported from pupal stages of *C. megacephala* in a much higher percentage [29]. Results from this analysis have shown that the bacterial composition is related to the type of fly species. For example, *Pseudomonas*,* Paracoccus*,* Enterococcus*, and *Luteimonas* bacterial taxa were detected only from *L. cuprina* samples with high abundances. While *Providencia*,* Fusobacterium*,* Escherichia-Shigella*,* Proteus*,* Collinsella*, and *Bacillus* bacterial taxa were recorded in high abundances from only the *P. clausa* samples from KMC, Gambia. Most bacterial taxa found in *P. clausa* are similar to bacterial taxa found in *M. domestica* of this study. Other *M. domestica* studies have found a similar bacterial composition [33], with the most predominant bacterial taxa; therefore, a thorough comparative analysis of the gut contents of these two flies is needed. These bacterial taxa were isolated from urban flies, and flies from this study were collected from urban settings in both cities, which can partly explain the similarity in bacterial composition between the two studies. Some of the variation observed in the presence of certain bacterial taxa at the genus level across the different fly species in this study could also be due to the fact that synanthropic flies share food sources. The observed differences in bacterial abundance between KMC and Changsha samples may reflect differences in sanitation and waste-management practices, available decomposing substrate, and dominant feeding sources in both cities [24].

Most of the reported bacterial taxa observed in the samples from our study were classified at the genus level. Several unclassified bacterial species were reported. This could indicate new species from these genera, and the fact that the Silva reference database has no reference sequences that are similar to those from this study [24, 35].

Our study also revealed that despite some samples being from the same species and collected from the same country and location, the month the samples were collected (sampling collection time) shows overwhelming variations in the bacterial composition of these flies, just as suggested in a previous study [33].

### Potential bacterial pathogens

Potential pathogenic bacterial taxa have been isolated from the midgut of these flies from both cities. These pathogenic taxa are implicated as disease-causing agents in humans and animals and therefore warrant appropriate attention for public health concerns. *Empedobacter falsenii*, isolated from *M. domestica*, is an opportunistic Gram-negative pathogen that causes several infections in humans, especially immunocompromised individuals [36]. This bacterium has been isolated from various clinical samples (stool, ear pus, urine) and has also been found to cause bacteremia and wound infections [37–39]. In this study, the environmental conditions, such as the sample collection sites in both cities, were approximately 300–400 m near public hospitals, which could explain why this bacterium was detected, as previous studies have implicated it in carrying multi-resistance genes and in causing nosocomial infections [40]. The bacterial composition due to fly collection-site variation is not the purpose of this study; however, it has been reported to cause significant variation in the bacterial composition of non-biting synanthropic flies elsewhere [41].

*Wohlfahrtiimonas chitiniclastica*, *Ignatzschineria indica*, and *Ignatzschineria ureiclastica* are fly-borne bacteria found in close association with humans [42]. These bacteria were found in all samples of our study, from both cities, but were most predominantly found in the KMC samples. The parasitic genera *Ignatzschineria* and *Wohlfahrtiimonas* have been isolated from the *Wohlfahrtia magnifica* fly and ever since have been found to cause human and animal myiasis— infection of open wounds (septicemia, and bacteremia) [43] which could lead to septic shock and eventually death [44]. The bacteria have been isolated from several clinical cases [45–48]. Snyder and colleagues, for example, recovered these bacteria from barrowing larvae in the toes of an 82-year-old patient [49]. Our findings further confirm that these bacteria are fly-associated and underscore the need for proper fly-control strategies, particularly around healthcare facilities. These bacteria are associated mainly with sarcophagid flies [50], which contrasts with our findings, where they were detected in calliphorids, muscids, and the picture-winged Ulidiidae. In our study, these bacterial species were detected in higher relative abundance in KMC than in Changsha samples, although environmental parameters were not recorded in either city [51]. *Wohlfahrtiimonas chitiniclastica* may, in addition to its pathogenic role, also degrade chitin, a major component of the insect skeleton [52]. This could explain its detection in all the samples from both cities at relatively high abundance.

We equally detected the pathogenic bacteria (*Weissella ceti*) that cause infections in rainbow trout, fish, and whale, as previously reported [53–55]. The bacterium was found mainly in the Changsha samples. The high abundance of this bacterium in the Changsha sample may reflect differences in feeding substrates between the two cities, which needs further monitoring.

The presence of *Corynebacterium variable* in all the flies from both countries suggested its association with the digestion and degradation of lipids and the nutrient cycle in flies [56], and its ability to utilize proteins and lipids in microbe-rich surface habitats is consistent with a role for *Corynebacterium* in processing the decomposing organic substrates that flies feed on, and could explain its detection in all the flies sampled in this study. However, it is worth noting that some species of the genus *Corynebacterium* are pathogenic to both humans and animals. *Corynebacterium ulceribovis* and *Corynebacterium humireducens* are Gram-positive bacteria from a genus recognized for animal-associated zoonotic pathogens. *C. ulceribovis* was first isolated from the ulcerated skin of a cow’s udder in Germany [57, 58]. *C. variabile* was originally isolated from the surface of smear-ripened cheeses, where it contributes to flavor and texture development through casein hydrolysis, fatty-acid degradation, and the catabolism of organic acids such as lactate and propionate, and *C. humireducens* was originally recovered from compost. Bacteria of the genus *Corynebacterium* were detected in all four fly species in this study, but these two species in particular were detected exclusively in *P. clausa* from KMC, Gambia. Their presence in this fly is notable because non-biting synanthropic flies are recognized as mechanical carriers of pathogens of veterinary relevance [59–61].

These findings should be interpreted in light of the limitations of this study. Biological replication was modest (three pooled samples per fly species per site, with each pool comprising 20 individuals), which is limited within-group statistical power and masks individual-level variation. The sampling months did not fully overlap between countries (August-October in KMC and September-November in Changsha); therefore, the inter-city signal was partly confounded by temporal variation. Environmental parameters (ambient temperature, substrate type, and sanitation indices) were not taken into account during sampling.

## Conclusion

This study explored the bacterial composition of the midgut of non-biting synanthropic flies from two countries. This comparative study not only addresses critical gap in understanding geographic microbial variation in these flies but also reported several bacterial genera with recognized veterinary importance, including *Wohlfahrtiimonas*,* Ignatzschineria*,* Vagococcus*,* Weissella*, and *Corynebacterium*, with city- and species-specific abundance patterns providing insights on species and geography-associated variation in microbial composition with observed city-specific abundance patterns at the genus level. Results from this study also provide the first reference data on the bacterial composition of *P. clausa* in the literature. Extensive pathogen isolation studies over a longer study duration are needed to confirm the viability and transmission potential of bacterial taxa identified in this study. We call for an in-depth characterization of the vector potential of *P. clausa* from the KMC, Gambia.

## Methods

### Fly sampling

Adult non-biting synanthropic flies were sampled between August and November 2023 in Kanifing Municipal Council (KMC) (Latitude: 13.45°N, Longitude: 16.68°W), Gambia, and Changsha City (Latitude: 28.23°N, Longitude: 112.94°E), China. We chose these two cities for two main reasons. The first is that they have very different sanitation and waste-management systems. Changsha operates a regulated, source-segregated municipal waste system, while KMC relies on a largely uncontrolled and informal one. This, in turn, affects the kind of decomposing material flies can feed on and how easily they can access it. Secondly, similar fly species were present in both cities, making cross-species comparison possible because different fly species have been reported to differ in gut bacterial composition. These factors have been shown to shape the gut microbiome of synanthropic flies, which enables a useful contrast in which a species-by-geography comparison was made.

Flies were collected at both cities around the main local market and 300–400 m away from the public hospital using commercial ranch-baited fly traps (manufactured in China). On each sampling day, one trap was deployed at each location and retrieved after a 9-hour deployment period. In each month’s collection, a new trap was used on each sampling day to avoid cross-contamination across sampling visits. Sampling was conducted 3 times per month for three consecutive months. After sorting and morphological identification, the three most abundant flies were selected for bacterial composition analysis in addition to the newly collected *P. clausa* flies from KMC, and 20 individuals of the same species were pooled into a single sample per month. This design yielded 21 pooled samples in total, with 6 traps in each month, amounting to 18 traps per country (18 trap-days in total). Nine from Changsha (three monthly pools each of *C. megacephala*, *L. cuprina*, and *M. domestica*) and 12 from KMC (three monthly pools each of *C. megacephala*, *L. cuprina*, *M. domestica*, and *P. clausa*. A total of 420 individual flies were analyzed. Sample identifiers and the corresponding species, city, and month for each pool are listed in Supplementary File 1, Table 1. Photographs of the trap setup at the representative sampling site in each city are provided as Supplementary Fig. 1.

A stereomicroscope (Motic SMZ-171) based on morphological diagnostic characteristics and region-wide taxonomic keys was used for species identification [62–65] and was confirmed by Sanger sequencing to ensure accuracy for taxonomic purposes. Convergence between morphological diagnostic features and molecular sequence measurements ensured reliable assignment of the specimens at the species level. Subspecies-level differentiation was not performed, as molecular confirmation provides sufficient resolution for species delimitation in the tested taxa.

Upon collection, flies from each trap were transferred into sterile plastic specimen bags at the sampling site and promptly placed in field coolers containing dry ice to ensure low temperatures during transport to the laboratory. The samples from Changsha were directly transported to the Meng Medical Entomology Laboratory, Department of Medical Parasitology, School of Basic Medical Sciences, Central South University, Changsha, China. Conversely, the KMC samples were initially transported and stored at the Medical Research Council Unit, The Gambia (MRCG) and subsequently shipped to the Meng Medical Entomology Laboratory in Falcon tubes, which were tightly sealed with parafilm and packed with silica gel desiccants to maintain dryness during international transport. All specimens from both cities were stored in − 80 °C until DNA extraction and subsequent molecular analyses of all samples were conducted at the Meng Medical Entomology Laboratory. All sequencing data generated in this study, including metabarcoding datasets, are publicly available in GenBank under the SRA accession number PRJNA1077112.

### DNA extraction and PCR

Pooled flies were surface sterilized with 75% ethanol and 10% bleach (0.8% sodium hypochlorite) for 60 s. This was followed by two rinses in autoclaved distilled water, each lasting 5 min. The samples were subsequently rinsed in sterile phosphate-buffered saline (PBS) for a duration of 15 min to avoid cross-contamination [66]. The midgut samples were crushed using a motorized hand homogenizer to form fly homogenates, and total genomic DNA was extracted from adult flies using the TIANamp Genomic DNA Kit (Cat. no. 4992199/4992254; TIANGEN, China) according to the manufacturer’s instructions. To recover the maximum amount of DNA, each sample was subjected to elution twice using 50 µL of elution buffer (TE buffer). DNA concentration was quantified using a spectrophotometer, and integrity was verified by 1% agarose gel electrophoresis. The midgut of 420 flies was pooled (*n* = 20/sample) by species (*C. megacephala*,* L. cuprina*,* M. domestica* from both cities and *P. clausa* from KMC, Gambia).

The V3-V4 hyper-variable region of the bacterial 16S rDNA gene of adult non-biting synanthropic flies was sequenced on the Illumina NovaSeq6000 platform using the primer 341F: 5’-ACTCCTACGGGAGGCAGCA-3’; 805R: 5’-GGACTACHVGGGTWTCTAAT-3’ [67, 68]. PCR was performed in triplicate in a 25 µL reaction mixture following the instructions of KFX-101 (TOBOYO, Tokyo). Purified amplicons from different samples were pooled in equimolar amounts and sequenced by BioMarker Technologies Co., Ltd. (Beijing, China) (www.biomarker.com.cn).

### Bioinformatics and statistical analysis

Raw data were filtered and processed by Trimmomatic (version 0.33) [69] to generate high-quality reads. The primer sequences were identified and removed by cutadapt (version 1.9.1) [70]. High-quality reads were clustered into OTUs by USEARCH (version 10.0) [71] and followed by chimera removal using UCHIME (version 8.1) [72]. Operational taxonomic units (OTUs) were defined as features in the analysis. Taxonomic annotation was performed at the OTU level. Microbial community composition at the phylum, class, order, family, genus, and species levels was revealed using taxonomic annotation in the Silva 138 Database [73]. Relative abundance was used to describe how much each bacterial taxon contributed to the total bacterial community at a given taxonomic level. It was calculated from the proportion of sequencing reads or OTUs assigned to each taxon within a sample or sample group and then expressed as a percentage for visualization and comparison.

Alpha and Beta diversities were evaluated by QIIME2 (version 2020.06) [74], combined with the algorithms R package, all statistical analyses were performed in R using the Vegan package [75] and ggplot2 was used for data visualization [76]. A bacterial diversity analysis of samples from the two cities of the two countries was compared using the Wilcoxon rank-sum test. The differences were then statistically quantified (*P* ≤ 0.05) using PERMANOVA (permutational multivariate analysis of variance) [77]. A spreadsheet of all analyzed taxonomic OTU counts per sample was also generated through Microsoft Excel to allow further comparison and is attached as supplementary file 2–6.

## Supplementary Information

Supplementary Material 1. Supplementary file_1_Table 1: Sample ID mapping table for the 21 pooled fly midgut samples analyzed in this study.

Supplementary Material 2. Supplementary file 2: Relative abundances (%) of phylum, family, genus across all samples.

Supplementary Material 3. Supplementary file 3: Relative abundances (%) of phylum, family, genus of *C. megacephala*.

Supplementary Material 4. Supplementary file 4: Relative abundance (%) of phylum, family, genus of *L. cuprina*.

Supplementary Material 5. Supplementary file 5: Relative abundance (%) of phylum, family, genus of *M. domestica*.

Supplementary Material 6. Supplementary file 6: Relative abundance (%) of phylum, family, genus of *P clausa*.

Supplementary Material 7. Supplementary Figure 1: Photographs of the trap setup at the sampling site in Changsha and Kanifing Municipal Council.

## Data Availability

The datasets generated and/or analyzed during the current study are available in GenBank under SRA accession: **PRJNA1077112**.

## Abbreviations

rDNA: Ribosomal DNA
PCA: Principal Component Analysis
PCoA: Principal Coordinate Analysis
KMC: Kanifing Municipal Council (in this study, KMC is used to refer to the area from Kanifing Municipal Council to Brikama Marakissa)
PERMANOVA: permutational multivariate analysis of variance
ChSC: *Chrysomya megacephala* September Changsha
ChOC: *Chrysomya megacephala* October Changsha
ChNC: *Chrysomya megacephala* November Changsha
ChAG: *Chrysomya megacephala* August Gambia
ChSG: *Chrysomya megacephala* September Gambia
ChOG: *Chrysomya megacephala* October Gambia
LSC: *Lucilia cuprina* September Changsha
LOC: *Lucilia cuprina* October Changsha
LNC: *Lucilia cuprina* November Changsha
LAG: *Lucilia cuprina* August Gambia
LSG: *Lucilia cuprina* September Gambia
LOG: *Lucilia cuprina* October Gambia
MSC: *Musca domestica* September Changsha
MOC: *Musca domestica* October Changsha
MNC: *Musca domestica* November Changsha
MAG: *Musca domestica* August Gambia
MSG: *Musca domestica* September Gambia
MOG: *Musca domestica* October Gambia
NDS1: New Species - *Physiphora* species (*P. clausa*) August
NDS2: New Species - *Physiphora* species (*P. clausa*) September
NDS3: New Species - *Physiphora* species (*P. clausa*) October

## References

[1] Stoffolano JG. Jr. Synanthropic flies-A review including how they Obtain nutrients, along with pathogens, store them in the crop and mechanisms of transmission. Insects. 2022;13(9). 10.3390/insects13090776. 10.3390/insects13090776 PMC9500719 36135477

[2] Cadavid-Sanchez IC, Amat E, Gomez-Piñerez LM. Enterobacteria isolated from synanthropic flies (Diptera, Calyptratae) in Medellín, Colombia. Caldasia. 2015;37(2):319–32. 10.15446/caldasia.v37n2.53594.

[3] Graczyk TK, Knight R, Gilman RH, Cranfield MR. The role of non-biting flies in the epidemiology of human infectious diseases. Microbes Infect. 2001;3(3):231–5. 10.1016/S1286-4579(01)01371-5. 11358717 10.1016/s1286-4579(01)01371-5

[4] Adenusi AA, Adewoga TOS. Human intestinal parasites in non-biting synanthropic flies in Ogun State, Nigeria. Travel Med Infect Dis. 2013;11(3):181–9. 10.1016/j.tmaid.2012.11.003. 23290716 10.1016/j.tmaid.2012.11.003

[5] Liu Y, Chen Y, Wang N, Qin H, Zhang L, Zhang S. The global prevalence of parasites in non-biting flies as vectors: A systematic review and meta-analysis. Parasites Vectors. 2023;16(1):1–20. 10.1186/s13071-023-05650-2. 36691084 10.1186/s13071-023-05650-2 PMC9872427

[6] Chaiwong T, Srivoramas T, Sukontason K, Sanford MR, Moophayak K, Sukontason KL. Survey of the synanthropic flies associated with human habitations in Ubon Ratchathani province of northeast Thailand. J Parasitol Res. 2012;2012. 10.1155/2012/613132. 10.1155/2012/613132 PMC3425801 22934155

[7] Dawaye DA, Djaouda M, Fils EMB. [Diversity of synanthropic flies and their potential for transmitting diarrheal diseases in Maroua (Far North- Cameroon)]. Pan Afr Med J. 2021;38:410. 10.11604/pamj.2021.38.410.24687. 34381554 10.11604/pamj.2021.38.410.24687 PMC8325447

[8] Meng F, Ren L, Wang Z, Deng J, Guo Y, Chen C, et al. Identification of forensically important blow flies (Diptera: Calliphoridae) in China based on COI. J Med Entomol. 2017;54(5):1193–200. 10.1093/jme/tjx105. 28535279 10.1093/jme/tjx105

[9] Rombot DV, Semuel MY, Kanan M. Bacterial species associate on the body surface of *Musca domestica* L from various habitats based on 16S rRNA sequencing. J Pure Appl Microbiol. 2023;17(3). 10.22207/JPAM.17.3.10.

[10] Jallow BJ, Gassara G, Bajinka O, Luo Y, Liu M, Cai J, et al. Human myiasis in Sub-Saharan Africa: a systematic review. PLoS Negl Trop Dis. 2024;18(3):e0012027. 10.1371/journal.pntd.0012027. 38547087 10.1371/journal.pntd.0012027 PMC10977789

[11] Liu Y, Chen Y, Wang N, Qin H, Zhang L, Zhang S. The global prevalence of parasites in non-biting flies as vectors: a systematic review and meta-analysis. Parasites vectors. 2023;16(1):25. 10.1186/s13071-023-05650-2. 36691084 10.1186/s13071-023-05650-2 PMC9872427

[12] Sukontason K, Bunchoo M, Khantawa B, Piangjai S, Methanitikorn R, Rongsriyam Y. Mechanical carrier of bacterial enteric pathogens by *Chrysomya megacephala* (Diptera: Calliphoridae) in Chiang Mai, Thailand. Southeast Asian J Trop Med Public Health. 2000;31:157–61. https://europepmc.org/article/med/11414447. 11414447

[13] Capone D, Adriano Z, Cumming O, Irish SR, Knee J, Nala R, et al. Urban onsite sanitation upgrades and synanthropic flies in Maputo, Mozambique: effects on enteric pathogen infection risks. Environ Sci Technol. 2022;57(1):549–60. 10.1021/acs.est.2c06864. 36516327 10.1021/acs.est.2c06864 PMC9835884

[14] Onwugamba FC, Fitzgerald JR, Rochon K, Guardabassi L, Alabi A, Kühne S, et al. The role of ‘filth flies’ in the spread of antimicrobial resistance. Travel Med Infect Dis. 2018;22:8–17. 10.1016/j.tmaid.2018.02.007. 29482014 10.1016/j.tmaid.2018.02.007

[15] Adenusi AA, Adewoga TO. Studies on the potential and public health importance of non-biting synanthropic flies in the mechanical transmission of human enterohelminths. Trans R Soc Trop Med Hyg. 2013;107(12):812–8. 10.1093/trstmh/trt095. 24128876 10.1093/trstmh/trt095

[16] Förster M, Sievert K, Messler S, Klimpel S, Pfeffer K. Comprehensive study on the occurrence and distribution of pathogenic microorganisms carried by synanthropic flies caught at different rural locations in Germany. J Med Entomol. 2009;46(5):1164–6. 10.1603/033.046.0526. 19769050 10.1603/033.046.0526

[17] AbdAllah OR, Gabre RM, Mohammed SA, Korayem AM, Hussein HE, Ahmad AA. Evaluating the role of synanthropic filth flies in the transmission of zoonotic parasites: field and laboratory evidence from different animal rearing sites in Upper Egypt with focus on Cryptosporidium spp. BMC Vet Res. 2025;21:188. 10.1186/s12917-025-04627-w. 40114149 10.1186/s12917-025-04627-w PMC11924607

[18] Bahrndorff S, De Jonge N, Skovgård H, Nielsen JL. Bacterial communities associated with houseflies (*Musca domestica L.*) sampled within and between farms. PLoS ONE. 2017;12(1):e0169753. 10.1371/journal.pone.0169753. 28081167 10.1371/journal.pone.0169753 PMC5232358

[19] Querejeta M, Hervé V, Perdereau E, Marchal L, Herniou EA, Boyer S, et al. Changes in bacterial community structure across the different life stages of black soldier fly (*Hermetia illucens*). Microb Ecol. 2023;86(2):1254–67. 10.1007/s00248-022-02146-x. 36434303 10.1007/s00248-022-02146-x

[20] Bell M, Irish S, Schmidt WP, Nayak S, Clasen T, Cameron M. Comparing trap designs and methods for assessing density of synanthropic flies in Odisha, India. Parasites Vectors. 2019;12(1):75. 10.1186/s13071-019-3324-z. 30732628 10.1186/s13071-019-3324-z PMC6367737

[21] Junqueira ACM, Ratan A, Acerbi E, Drautz-Moses DI, Premkrishnan BN, Costea PI, et al. The microbiomes of blowflies and houseflies as bacterial transmission reservoirs. Sci Rep. 2017;7(1):16324. 10.1038/s41598-017-16353-x. 29176730 10.1038/s41598-017-16353-x PMC5701178

[22] Gupta AK, Nayduch D, Verma P, Shah B, Ghate HV, Patole MS, et al. Phylogenetic characterization of bacteria in the gut of house flies (*M usca domestica L*). FEMS Microbiol Ecol. 2012;79(3):581–93. 10.1111/j.1574-6941.2011.01248.x. 22092755 10.1111/j.1574-6941.2011.01248.x

[23] Mason CJ, Auth J, Geib SM. Gut bacterial population and community dynamics following adult emergence in pest tephritid fruit flies. Sci Rep. 2023;13(1):13723. 10.1038/s41598-023-40562-2. 37607978 10.1038/s41598-023-40562-2 PMC10444893

[24] Park R, Dzialo MC, Spaepen S, Nsabimana D, Gielens K, Devriese H, et al. Microbial communities of the house fly *Musca domestica* vary with geographical location and habitat. Microbiome. 2019;7:1–12. 10.1186/s40168-019-0748-9. 31699144 10.1186/s40168-019-0748-9 PMC6839111

[25] Wang X, Gao Q, Wang W, Wang X, Lei C, Zhu F. The gut bacteria across life stages in the synanthropic fly *Chrysomya megacephala*. BMC Microbiol. 2018;18:1–8. 10.1186/s12866-018-1272-y. 30305025 10.1186/s12866-018-1272-y PMC6180576

[26] Zhao Z, Dong H, Wang R, Zhao W, Chen G, Li S et al. Genotyping and subtyping *Cryptosporidium parvum* and *Giardia duodenalis* carried by flies on dairy farms in Henan, China. Parasites & Vectors. 2014;7:1–6. http://www.parasitesandvectors.com/content/7/1/190. 10.1186/1756-3305-7-190 PMC4005625 24742088

[27] Neupane S, Saski C, Nayduch D. House fly larval grazing alters dairy cattle manure microbial communities. BMC Microbiol. 2021;21(1):346. 10.1186/s12866-021-02418-5. 34911456 10.1186/s12866-021-02418-5 PMC8672618

[28] de Jonge N, Michaelsen TY, Ejbye-Ernst R, Jensen A, Nielsen ME, Bahrndorff S, et al. Housefly (*Musca domestica L.*) associated microbiota across different life stages. Sci Rep. 2020;10(1):7842. 10.1038/s41598-020-64704-y. 32398740 10.1038/s41598-020-64704-y PMC7217826

[29] Xu W, Wang Y, Wang Y-h, Zhang Y-n, Wang J-f. Diversity and dynamics of bacteria at the *Chrysomya megacephala* pupal stage revealed by third-generation sequencing. Sci Rep. 2022;12(1):2006. 10.1038/s41598-022-06311-7. 35132164 10.1038/s41598-022-06311-7 PMC8821589

[30] Pileggi MT, Chase JR, Shu R, Teng L, Jeong KC, Kaufman PE, et al. Prevalence of field-collected house flies and stable flies with bacteria displaying cefotaxime and multidrug resistance. J Med Entomol. 2021;58(2):921–8. 10.1093/jme/tjaa241. 33210705 10.1093/jme/tjaa241

[31] Iancu L, Angelescu IR, Paun VI, Henríquez-Castillo C, Lavin P, Purcarea C. Microbiome pattern of *Lucilia sericata* (Meigen)(Diptera: Calliphoridae) and feeding substrate in the presence of the foodborne pathogen Salmonella enterica. Sci Rep. 2021;11(1):15296. 10.1038/s41598-021-94761-w. 34315964 10.1038/s41598-021-94761-w PMC8316364

[32] Zurek K, Nayduch D. Bacterial associations across house fly life history: evidence for transstadial carriage from managed manure. J Insect Sci. 2016;16(1). 10.1093/jisesa/iev156. 10.1093/jisesa/iev156 PMC4725258 26798138

[33] Neupane S, Nayduch D. Effects of habitat and sampling time on bacterial community composition and diversity in the gut of the female house fly, Musca domestica Linnaeus (Diptera: Muscidae). Med Vet Entomol. 2022;36(4):435–43. 10.1111/mve.12581. 35599244 10.1111/mve.12581

[34] Gasz N, Geary MJ, Doggett SL, Harvey M. Bacterial association observations in *Lucilia sericata* and *Lucilia cuprina* organs through 16S rRNA gene sequencing. Appl Microbiol Biotechnol. 2021;105:1091–106. 10.1007/s00253-020-11026-8. 33415370 10.1007/s00253-020-11026-8

[35] Meng F, Liu Z, Sun J, Kong D, Wang Y, Tong X, et al. Insights into gut microbiota communities of Poecilobdella manillensis, a prevalent Asian medicinal leech. J Appl Microbiol. 2022;133(3):1402–13. 10.1111/jam.15514. 35262268 10.1111/jam.15514

[36] Olowo-Okere A, Ibrahim YKE, Olayinka BO, Mohammed Y, Nabti LZ, Lupande-Mwenebitu D, et al. Genomic features of an isolate of *Empedobacter falsenii* harbouring a novel variant of *metallo-β-lactamase*, bla(EBR-4) gene. Infect Genet Evol. 2022;98:105234. 10.1016/j.meegid.2022.105234. 35121093 10.1016/j.meegid.2022.105234

[37] Traglia GM, Dixon C, Chiem K, Almuzara M, Barberis C, Montaña S, et al. Draft genome sequence of *Empedobacter* (Formerly Wautersiella) *falsenii comb.* nov. Wf282, a strain isolated from a cervical neck abscess. Genome Announcements. 2015;3(2). 10.1128/genomeA.00235-15. 10.1128/genomeA.00235-15 PMC4384494 25838490

[38] Van der Velden LB, de Jong AS, de Jong H, de Gier RP, Rentenaar RJ. First report of a Wautersiella falsenii isolated from the urine of an infant with pyelonephritis. Diagn Microbiol Infect Dis. 2012;74(4):404–5. 10.1016/j.diagmicrobio.2012.08.008. 22999333 10.1016/j.diagmicrobio.2012.08.008

[39] Martinez V, Matabang MA, Miller D, Aggarwal R, LaFortune A. First case report on Empedobacter falsenii bacteremia. IDCases. 2023;33:e01814. 10.1016/j.idcr.2023.e01814. 37645528 10.1016/j.idcr.2023.e01814 PMC10461116

[40] Collins C, Almuzara M, Saigo M, Montaña S, Chiem K, Traglia G, et al. Whole-genome analysis of an extensively drug-resistance *Empedobacter falsenii* strain reveals distinct features and the presence of a novel metallo-ß-lactamase (EBR-2). Curr Microbiol. 2018;75(8):1084–9. 10.1007/s00284-018-1498-9. 29687150 10.1007/s00284-018-1498-9

[41] Neupane S, Talley JL, Taylor DB, Nayduch D. Bacterial communities and prevalence of antibiotic resistance genes carried within house flies (Diptera: Muscidae) associated with beef and dairy cattle farms. J Med Entomol. 2023;60(6):1388–97. 10.1093/jme/tjad112. 37612042 10.1093/jme/tjad112 PMC11502952

[42] Deel HL, Montoya S, King K, Emmons AL, Huhn C, Lynne AM, et al. The microbiome of fly organs and fly-human microbial transfer during decomposition. Forensic Sci Int. 2022;340:111425. 10.1016/j.forsciint.2022.111425. 36087369 10.1016/j.forsciint.2022.111425

[43] DiFranza LT, Annavajhala MK, Uhlemann A-C, Green DA. The brief case: a maggot mystery—*Ignatzschineria* larvae sepsis secondary to an infested wound. J Clin Microbiol. 2021;59(3):02279–20. 10.1128/jcm. 10.1128/JCM.02279-20 PMC8106707 33826527

[44] Chavez JA, Alexander AJ, Balada-Llasat JM, Pancholi P. A case of *Wohlfahrtiimonas chitiniclastica* bacteremia in continental United States. JMM Case Rep. 2017;4(12). 10.1099/jmmcr.0.005134. 10.1099/jmmcr.0.005134 PMC5857366 29568530

[45] Reed K, Reynolds SB, Smith C, Smith CM. The first case of *Ignatzschineria ureiclastica*/larvae in the United States presenting as a myiatic wound infection. Cureus. 2021;13(4). 10.7759/cureus.14518. 10.7759/cureus.14518 PMC8126268 34007769

[46] Fear T, Richert Q, Levesque J, Walkty A, Keynan Y. *Ignatzschineria indica* bloodstream infection associated with maggot infestation of a wound in a patient from Canada. J Association Med Microbiol Infect Disease Can. 2020;5(3):193–200. 10.3138/jammi-2019-0027. 10.3138/jammi-2019-0027 PMC9608726 36341319

[47] Le Brun C, Gombert M, Robert S, Mercier E, Lanotte P. Association of necrotizing wounds colonized by maggots with *Ignatzschineria*-associated septicemia. Emerg Infect Dis. 2015;21(10):1881–3. 10.3201/eid2110.150748. 26402740 10.3201/eid2110.150748 PMC4593450

[48] de Dios A, Jacob S, Tayal A, Fisher MA, Dingle TC, Hamula CL. First report of *Wohlfahrtiimonas chitiniclastica* isolation from a patient with cellulitis in the United States. J Clin Microbiol. 2015;53(12):3942–4. 10.1128/jcm.01534-15. 26378273 10.1128/JCM.01534-15 PMC4652122

[49] Snyder S, Singh P, Goldman J. Emerging pathogens: a case of *Wohlfahrtiimonas chitiniclastica* and *Ignatzschineria indica* bacteremia. IDCases. 2020;19:e00723. 10.1016/j.idcr.2020.e00723. 32123664 10.1016/j.idcr.2020.e00723 PMC7037535

[50] Gupta AK, Dharne MS, Rangrez AY, Verma P, Ghate HV, Rohde M, et al. *Ignatzschineria indica* sp. nov. and *Ignatzschineria ureiclastica* sp. nov., isolated from adult flesh flies (Diptera: Sarcophagidae). Int J Syst Evol MicroBiol. 2011;61(6):1360–9. 10.1099/ijs.0.018622-0. 20584814 10.1099/ijs.0.018622-0

[51] Toth EM, Schumann P, Borsodi AK, Keki Z, Kovacs AL, Marialigeti K. *Wohlfahrtiimonas chitiniclastica* gen. nov., sp. nov., a new gammaproteobacterium isolated from Wohlfahrtia magnifica (Diptera: Sarcophagidae). Int J Syst Evol MicroBiol. 2008;58(4):976–81. 10.1099/ijs.0.65324-0. 18398205 10.1099/ijs.0.65324-0

[52] Schröttner P, Rudolph W, Damme U, Lotz C, Jacobs E, Gunzer F. *Wohlfahrtiimonas chitiniclastica*: current insights into an emerging human pathogen. Epidemiol Infect. 2017;145(7):1292–303. 10.1017/S0950268816003411. 28162132 10.1017/S0950268816003411 PMC9203347

[53] Figueiredo HC, Soares SC, Pereira FL, Dorella FA, Carvalho AF, Teixeira JP, et al. Comparative genome analysis of *Weissella ceti*, an emerging pathogen of farm-raised rainbow trout. BMC Genomics. 2015;16:1095. 10.1186/s12864-015-2324-4. 26694728 10.1186/s12864-015-2324-4 PMC4687380

[54] Liu JY, Li AH, Ji C, Yang WM. First description of a novel *Weissella* species as an opportunistic pathogen for rainbow trout Oncorhynchus mykiss (Walbaum) in China. Vet Microbiol. 2009;136(3–4):314–20. 10.1016/j.vetmic.2008.11.027. 19321272 10.1016/j.vetmic.2008.11.027

[55] Björkroth KJ, Schillinger U, Geisen R, Weiss N, Hoste B, Holzapfel WH, et al. Taxonomic study of Weissella confusa and description of Weissella cibaria sp. nov., detected in food and clinical samples. Int J Syst Evol MicroBiol. 2002;52(Pt 1):141–8. 10.1099/00207713-52-1-141. 11837296 10.1099/00207713-52-1-141

[56] Yang S, Xie J, Hu N, Liu Y, Zhang J, Ye X, et al. Bioconversion of gibberellin fermentation residue into feed supplement and organic fertilizer employing housefly (*Musca domestica L*.) assisted by Corynebacterium variabile. PLoS ONE. 2015;10(5):e0110809. 10.1371/journal.pone.0110809. 25992605 10.1371/journal.pone.0110809 PMC4439168

[57] Schröder J, Maus I, Trost E, Tauch A. Complete genome sequence of *Corynebacterium variabile* DSM 44702 isolated from the surface of smear-ripened cheeses and insights into cheese ripening and flavor generation. BMC Genomics. 2011;12:545. 10.1186/1471-2164-12-545. 22053731 10.1186/1471-2164-12-545 PMC3219685

[58] Yassin AF, Lapidus A, Han J, Reddy T, Huntemann M, Pati A, et al. High quality draft genome sequence of *Corynebacterium ulceribovis* type strain IMMIB-L1395T (DSM 45146T). Stand Genomic Sci. 2015;10(1):50. 10.1186/s40793-015-0036-7. 26380638 10.1186/s40793-015-0036-7 PMC4572677

[59] Torky HA, Saad HM, Khaliel SA, Kassih AT, Sabatier JM, Batiha GE, et al. Isolation and molecular characterization of *Corynebacterium pseudotuberculosis*: association with proinflammatory cytokines in caseous lymphadenitis pyogranulomas. Anim (Basel). 2023;13(2). 10.3390/ani13020296. 10.3390/ani13020296 PMC9854522 36670836

[60] Ekman L, Bagge E, Nyman A, Persson Waller K, Pringle M, Segerman B. A shotgun metagenomic investigation of the microbiota of udder cleft dermatitis in comparison to healthy skin in dairy cows. PLoS ONE. 2020;15(12):e0242880. 10.1371/journal.pone.0242880. 33264351 10.1371/journal.pone.0242880 PMC7710049

[61] Amat E. Gomez-Piñerez lm. enterobacteria isolated from synanthropic flies (diptera, calyptratae) in medellín, colombia. caldasia. 2015. 10.15446/caldasia.v37n2.53594.

[62] Yang S-T, Kurahashi H, Shiao S-F. Keys to the blow flies of Taiwan, with a checklist of recorded species and the description of a new species of *Paradichosia Senior-White* (Diptera, Calliphoridae). ZooKeys. 2014;43457. 10.3897/zookeys.434.7540. 10.3897/zookeys.434.7540 PMC4141167 25152681

[63] Lutz L, Williams KA, Villet MH, Ekanem M, Szpila K. Species identification of adult African blowflies (Diptera: Calliphoridae) of forensic importance. Int J Legal Med. 2018;132(3):831–42. 10.1007/s00414-017-1654-y. 28849264 10.1007/s00414-017-1654-y PMC5919996

[64] Couri MS. A key to the Afrotropical genera of Muscidae (Diptera). Revista Brasileira de Zoologia. 2007;24:175–84. 10.1590/S0101-81752007000100022.

[65] Ji B-H, Park S-H, Moon T-Y. Pictorial identification key for blowflies (Diptera, Calliphoridae) of potential forensic importance in Korea. Korean J Legal Med. 2021;45(1):22–6. 10.7580/kjlm.2021.45.1.22.

[66] Hammer TJ, Dickerson JC, Fierer N. Evidence-based recommendations on storing and handling specimens for analyses of insect microbiota. PeerJ. 2015;3:e1190. 10.7717/peerj.1190. 26311208 10.7717/peerj.1190 PMC4548535

[67] Herlemann DP, Labrenz M, Jürgens K, Bertilsson S, Waniek JJ, Andersson AF. Transitions in bacterial communities along the 2000 km salinity gradient of the Baltic Sea. ISME J. 2011;5(10):1571–9. 10.1038/ismej.2011.41. 21472016 10.1038/ismej.2011.41 PMC3176514

[68] Klindworth A, Pruesse E, Schweer T, Peplies J, Quast C, Horn M, et al. Evaluation of general 16S ribosomal RNA gene PCR primers for classical and next-generation sequencing-based diversity studies. Nucleic Acids Res. 2013;41(1):e1–e. 10.1093/nar/gks808. 22933715 10.1093/nar/gks808 PMC3592464

[69] Bolger AM, Lohse M, Usadel B. Trimmomatic: a flexible trimmer for Illumina sequence data. Bioinformatics. 2014;30(15):2114–20. 10.1093/bioinformatics/btu170. 24695404 10.1093/bioinformatics/btu170 PMC4103590

[70] Martin M. Cutadapt removes adapter sequences from high-throughput sequencing reads. EMBnet J. 2011;17(1):10–. 10.14806/ej.17.1.200. 2.

[71] Edgar RC. Search and clustering orders of magnitude faster than BLAST. Bioinformatics. 2010;26(19):2460–1. 10.1093/bioinformatics/btq461. 20709691 10.1093/bioinformatics/btq461

[72] Edgar RC, Haas BJ, Clemente JC, Quince C, Knight R. UCHIME improves sensitivity and speed of chimera detection. Bioinformatics. 2011;27(16):2194–200. 10.1093/bioinformatics/btr381. 21700674 10.1093/bioinformatics/btr381 PMC3150044

[73] Quast C, Pruesse E, Yilmaz P, Gerken J, Schweer T, Yarza P, et al. The SILVA ribosomal RNA gene database project: improved data processing and web-based tools. Nucleic Acids Res. 2012;41(D1):D590–6. 10.1093/nar/gks1219. 23193283 10.1093/nar/gks1219 PMC3531112

[74] Bolyen E, Rideout JR, Dillon MR, Bokulich NA, Abnet CC, Al-Ghalith GA, et al. Reproducible, interactive, scalable and extensible microbiome data science using QIIME 2. Nat Biotechnol. 2019;37(8):852–7. 10.1038/s41587-019-0190-3. 31341288 10.1038/s41587-019-0209-9 PMC7015180

[75] Dixon P. VEGAN, a package of R functions for community ecology. J Veg Sci. 2003;14(6):927–30. 10.1111/j.1654-1103.2003.tb02228.x.

[76] Wickham H. ggplot2: Elegant graphics for data analysis. Springer Int Publishing Cham. 2016;17(3). 10.1080/15366367.2019.1565254.

[77] Anderson MJ. A new method for non-parametric multivariate analysis of variance. Austral Ecol. 2001;26(1):32–46. 10.1111/j.1442-9993.2001.01070.pp.x.

