# Supplementary material for: Comparative study on the diversity and abundance of bacterial composition in four non-biting synanthropic flies from The Gambia and China
Source: BMC Vet Res. 2026 Jun 15;22:492. doi: 10.1186/s12917-026-05620-7 (PMC13508449; doi:10.1186/s12917-026-05620-7)
Supplement: Supplementary file 1 [file 12917_2026_5620_MOESM7_ESM.pdf]

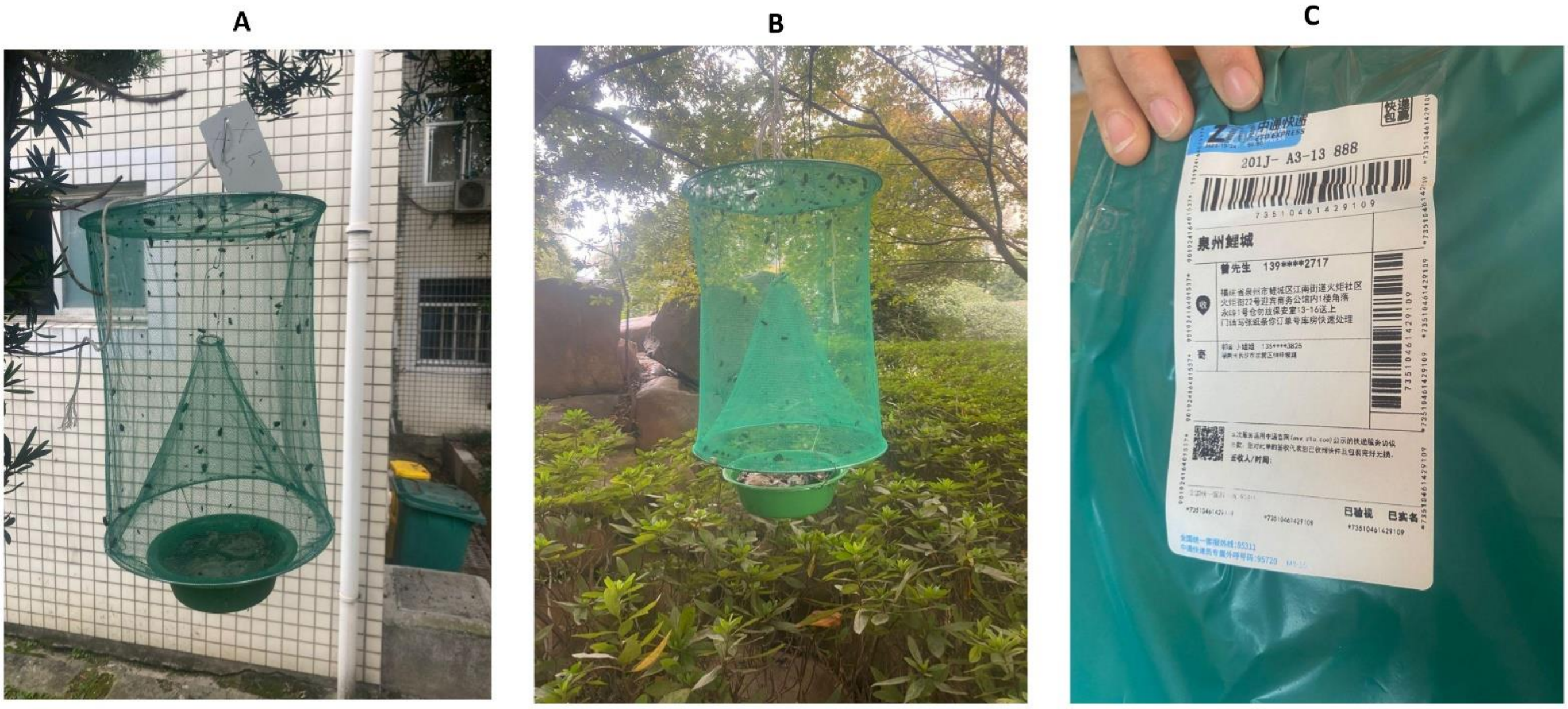

Supplementary Figure 1: A) Fly collection site in Changsha City, China. B) Fly collection site in Kanifing Municipal Council (KMC), The Gambia. C) Procurement and packaging details of the fly traps from the Taobao online marketplace, confirming the source and model of the "Ranch Trap" used for sample collection.
