# Supplementary material for: Comparative study on the diversity and abundance of bacterial composition in four non-biting synanthropic flies from The Gambia and China
Source: BMC Vet Res. 2026 Jun 15;22:492. doi: 10.1186/s12917-026-05620-7 (PMC13508449; doi:10.1186/s12917-026-05620-7)
Supplement: Supplementary file 2 [file 12917_2026_5620_MOESM1_ESM.docx]

Table 1: Sample ID mapping table

| Sample ID | Fly species | Month Collected | Country |
| --- | --- | --- | --- |
| ChSC | *Ch. megacephala* | Sep | Changsha |
| ChOC | *Ch. megacephala* | Oct | Changsha |
| ChNC | *Ch. megacephala* | Nov | Changsha |
| ChAG | *Ch. megacephala* | Aug | Gambia |
| ChSG | *Ch. megacephala* | Sep | Gambia |
| ChOG | *Ch. megacephala* | Oct | Gambia |
| LSC | *L. cuprina* | Sep | Changsha |
| LOC | *L. cuprina* | Oct | Changsha |
| LNC | *L. cuprina* | Nov | Changsha |
| LAG | *L. cuprina* | Aug | Gambia |
| LSG | *L. cuprina* | Sep | Gambia |
| LOG | *L. cuprina* | Oct | Gambia |
| MSC | *M. domestica* | Sep | Changsha |
| MOC | *M. domestica* | Oct | Changsha |
| MNC | *M. domestica* | Nov | Changsha |
| MAG | *M. domestica* | Aug | Gambia |
| MSG | *M. domestica* | Sep | Gambia |
| MOG | *M. domestica* | Oct | Gambia |
| NDS1 | *P. clausa* | Aug | Gambia |
| NDS2 | *P. clausa* | Sep | Gambia |
| NDS3 | *P. clausa* | Oct | Gambia |

Ch-*Chrysomya megacephala*, L- *Lucilia cuprina*, M-*Musca domestica*, NDS- *Physiphora clausa*, Aug- August, Sep-September, Oct- October, Nov- November, C-Changsha, G-Gambia
